# Azacitidine Post-Remission Therapy for Elderly Patients with AML: A Randomized Phase-3 Trial (QoLESS AZA-AMLE)

**DOI:** 10.3390/cancers15092441

**Published:** 2023-04-24

**Authors:** Esther Natalie Oliva, Anna Candoni, Prassede Salutari, Giuseppe A. Palumbo, Gianluigi Reda, Giuseppe Iannì, Giovanni Tripepi, Maria Cuzzola, Debora Capelli, Corrado Mammì, Caterina Alati, Maria Concetta Cannatà, Pasquale Niscola, Bianca Serio, Pellegrino Musto, Ernesto Vigna, Antonio Volpe, Lorella Maria Antonia Melillo, Maria Teresa Arcadi, Donato Mannina, Maria Elena Zannier, Roberto Latagliata

**Affiliations:** 1 U.O.C. Ematologia, Grande Ospedale Metropolitano Bianchi Melacrino Morelli, 89124 Reggio di Calabria, Italy; 2 Divisione Ematologia, P.O. Santa Maria della Misericordia, A.S.U.F.C di Udine, 33100 Udine, Italy; 3 Dipartimento Oncologico-Ematologico Ospedale Civile Spirito Santo Pescara, 65124 Pescara, Italy; 4 Dipartimento di Scienze Mediche Chirurgiche e Tecnologie Avanzate “G.F. Ingrassia”, University of Catania, 95123 Catania, Italy; 5 UOC Ematologia Università degli Studi di Milano, IRCCS Ospedale Maggiore Policlinico Milano, 20133 Milano, Italy; 6 Dielnet SRL, CRO Reggio Calabria, 89124 Reggio Calabria, Italy; 7 IFC-CNR Institute of Clinical Physiology Reggio Calabria, 89124 Reggio Calabria, Italy; 8 UOSD Tipizzazione Tissutale, Grande Ospedale Metropolitano Bianchi Melacrino Morelli, 89124 Reggio di Calabria, Italy; 9 Clinica di Ematologia Azienda Ospedaliera Universitaria, Ospedali Riuniti di Ancona, 60126 Ancona, Italy; 10 UOSD Medical Genetics, Great Metropolitan Hospital, 89124 Reggio Calabria, Italy; 11 U.O. di Ematologia, Ospedale Sant’Eugenio, 00144 Roma, Italy; 12 Dipartimento di Oncoematologia, AOU San Giovanni di Dio e Ruggi D’Aragona, 84125 Salerno, Italy; 13 Department of Precision and Translational Medicine with Ionian Area, “Aldo Moro” University School of Medicine, 70121 Bari, Italy; 14 Unit of Hematology and Stem Cell Transplantation, AOUC Policlinico, 70124 Bari, Italy; 15 U.O. di Ematologia, Ospedale L’Annunziata, 87100 Cosenza, Italy; 16 U.O. di Ematologia, Azienda Ospedaliera San Giuseppe Moscato, 83100 Avellino, Italy; 17 U.O.C. Ematologia e Trapianto di Cellule Staminali Emopoietiche, Policlinico Foggia Ospedaliero-Universitario, 71122 Foggia, Italy; 18 U.O. Farmacia, Grande Ospedale Metropolitano Bianchi Melacrino Morelli, 89124 Reggio di Calabria, Italy; 19 U.O.C. di Ematologia, Azienda Ospedaliera Papardo, 98158 Messina, Italy; 20 Divisione di Ematologia, Ospedale Belcolle, 01100 Viterbo, Italy

**Keywords:** acute myeloid leukemia, elderly, post-remission therapy, azacitidine

## Abstract

**Simple Summary:**

Azacitidine (AZA) is a hypomethylating agent with well-known antileukemic activity. Due to its favorable safety profile, AZA is widely used alone or in association with other drugs for the frontline treatment of patients with acute myeloid leukemia (AML) unfit for intensive chemotherapy. To date, only a few studies have used AZA as maintenance therapy during complete remission in patients with AML. In our phase-3 randomized multicenter trial, AZA improved disease-free survival (DFS) up to 2 and 5 years in patients aged >68 years compared to best supportive care (BSC). No patients died before leukemic relapse and no differences in patient-reported outcome measures between AZA and BSC patient groups were observed. The most frequent side effect seen in patients was low neutrophil count. In summary, AZA given as a post-remission therapy was found to provide benefit in AML patients aged >68 years.

**Abstract:**

This phase-3 randomized multicenter trial evaluated the efficacy of subcutaneous azacitidine (AZA) post-remission therapy vs. best supportive care (BSC) in elderly acute myeloid leukemia (AML) patients. The primary endpoint was the difference in disease-free survival (DFS) from complete remission (CR) to relapse/death. Patients with newly diagnosed AML aged ≥61 years received two courses of induction chemotherapy (“3+7” daunorubicin and cytarabine) followed by consolidation (cytarabine). At CR, 54 patients were randomized (1:1) to receive BSC (N = 27) or AZA (N = 27) at a dose of 50 mg/m^2^ for 7 days every 28 days and the dose increased after the 1st cycle to 75 mg/m^2^ for a further 5 cycles, followed by cycles every 56 days for 4.5 years. At 2 years, median DFS was 6.0 (95% CI: 0.2–11.7) months for patients receiving BSC vs. 10.8 months (95% CI: 1.9–19.6, *p* = 0.20) months for AZA. At 5 years, DFS was 6.0 (95% CI: 0.2–11.7) months in the BSC arm vs. 10.8 (95% CI: 1.9–19.6, *p* = 0.23) months in the AZA arm. Significant benefit was afforded by AZA on DFS at 2 and 5 years in patients aged >68 years (HR = 0.34, 95% CI: 0.13–0.90, *p* = 0.030 and HR = 0.37, 95% CI: 0.15–0.93, *p* = 0.034, respectively). No deaths occurred prior to leukemic relapse. Neutropenia was the most frequent adverse event. There were no differences in patient-reported outcome measures between study arms. In conclusion, AZA post-remission therapy was found to provide benefit in AML patients aged >68 years.

## 1. Introduction

The achievement of complete remission (CR) is an important milestone for patients with acute myeloid leukemia (AML) undergoing curative-intent therapy [1]. Advanced age, comorbidities, and biological aspects of leukemia in older adults [2] affect the ability of such patients to tolerate treatment and contribute to worse outcomes with lower rates of CR compared to younger patients [3,4].

Independent of their age, virtually all patients who achieve remission with induction therapy for AML will relapse within months, unless additional therapy is given [5]. Consequently, there has been long-standing interest in the use of lower-intensity maintenance therapies after completion of the intensive treatment phase, to prolong the duration of remission and increase survival and the likelihood of cure [6,7,8].

Azacitidine (AZA) is a hypomethylating agent with well-known antileukemic activity, widely used alone or in association with other drugs for the frontline treatment of AML patients unfit for intensive chemotherapy, due to its favorable safety profile [9,10,11,12,13]. A recent report tested AZA as maintenance therapy for 1-year treatment after CR achievement in elderly AML patients, showing an advantage in prolonging relapse-free survival (RFS) but not overall survival (OS) [14]. Moreover, in a placebo-controlled randomized clinical trial, which evaluated oral AZA formulation (CC-486) as a maintenance therapy in patients aged >55 years following induction, there were significantly longer OS and RFS in those receiving the investigational product [15].

The aim of the present randomized phase-3 trial was to test the efficacy and safety of long-term AZA maintenance compared to placebo in AML elderly patients who achieved their first CR after a homogeneous intensive induction and consolidation phase.

## 2. Materials and Methods

### 2.1. Study Design

This trial had a prospective, randomized, open-label, multicenter, national, phase-3 design. We estimated an enrolment of 135 patients with newly diagnosed AML to receive induction and consolidation chemotherapy to reach 54 patients in CR, randomized 1:1 to receive prospective AZA vs. Best Supportive Care (BSC) until relapse (Supplementary Materials Figure S1). Ethics committee approval and written informed consent was obtained from all patients. This study complies with ethical standards laid down in the 1975 Declaration of Helsinki and was registered at clinicaltrials.gov (NCT05188326) and in the EU Clinical Trials Register (2010-019710-24).

### 2.2. Patients

Patients were included according to the following criteria: age ≥ 61 years; newly diagnosed AML with >30% myeloid bone marrow (BM) blasts, either de novo or evolving from a myelodysplastic syndrome (MDS) not previously treated with chemotherapeutic agents; absence of central nervous system involvement; no contraindications for intensive chemotherapy, defined as: (a) prior congestive heart failure requiring treatment and/or left ventricular systolic ejection fraction below the normal range; (b) creatinine or bilirubin levels > 2-fold the upper limit of normal, except if AML-related; (c) ECOG performance status scale > 2; (d) uncontrolled severe infection.

### 2.3. Endpoints

The primary endpoint of this study was to evaluate the difference in disease-free survival (DFS) at 2 and 5 years between AZA and BSC arms. DFS was calculated from the date of achievement of CR to the date of first relapse (either AML or MDS recurrence) or death with censoring at the date of last contact if alive and disease-free.

Secondary endpoints were the number and length of hospitalizations in the post-remission period, OS at 2 and 5 years, and changes in quality of life (QoL) scores from diagnosis. The number and length of hospitalizations to assess the secondary endpoints did not include those required to receive the investigational treatment (AZA). OS was calculated from the date of achievement of CR to the date of death with censoring at the date of last contact if alive and disease-free. CR post induction was defined according to the following criteria [16]:

(a) BM contains <5% blasts, including monoblasts and promonocytes in M5 leukemia;
(b) BM cellularity of at least 20% with maturation of all cell lines;
(c) Absence of Auer rods;
(d) Absence of extramedullary leukemia;
(e) Absence of peripheral leukemic blasts;
(f) Hemoglobin levels ≥ 9 g/dL, absolute neutrophil count ≥ 1.5 × 10^9^/L, and platelet count ≥ 100 × 10^9^/L.

Subsequent CR evaluations were protocol-defined by BM evaluation every 6 months from randomization or at the discretion of the investigator.

Partial remission (PR) was defined as:

(a) BM contains 5–25% blasts, or <5% blasts in the presence of Auer rods;
(b) Absence of peripheral leukemic blasts.

If neither CR nor PR are reached, refractory AML is defined. 

QoL measures were obtained with QOL-E version 3 and EORTC QLQ-C30 version 3 questionnaires [17,18] at the following time points: baseline, at first induction cycle, after consolidation at randomization, post-remission cycle 2, 4, 7, and every 6 months after post-remission cycle 7. 

### 2.4. Induction and Consolidation Chemotherapy

Newly diagnosed AML patients underwent standard induction chemotherapy consisting of two courses of 3+7 with daunorubicin at a daily dosage of 40 mg/m^2^ for 3 days (days 1–3) in combination with 100 mg/m^2^ cytarabine per day as continuous intravenous infusion for 7 days (days 1–7). After the first and second cycle, BM aspirates were performed for response evaluation. 

After the second cycle of induction chemotherapy, patients in CR started consolidation therapy, which consisted of a 3 h infusion of 800 mg/m^2^ cytarabine given twice daily (days 1–3).

### 2.5. Post-Remission Therapy

Patients in CR after consolidation were randomly assigned to receive BSC or AZA therapy. AZA was administered according to the following regime: 50 mg/m^2^ subcutaneously for 7 days. If well tolerated during the first 28-day cycle (lack of treatment-emergent serious adverse events), the dose was increased for the following five cycles to 75 mg/m^2^, followed by 7-day administration every 56 days for a maximum of 4.5 years.

### 2.6. Bone Marrow Assessment

BM assessment for morphology and cytogenetics was performed in accordance with local laboratory procedures at diagnosis, after each induction cycle, after consolidation cycle, and every six months post the randomization phase until documented disease relapse. 

At the time the trial was written, measurable residual disease (MRD) was not a factor for prognostic and efficacy-response assessments; therefore, it was not mandatory to collect MRD data. However, MRD data from immunophenotype and mutational status were obtained from 40 evaluable cases with bone marrow samples collected at baseline, at randomization, and during the trial. MRD was evaluated in a single institution (Grande Ospedale Metropolitano Bianchi Melacrino Morelli, Reggio Calabria, Italy) by multiparameter flow cytometry (MFC)-MRD and real-time quantitative polymerase chain reaction (PCR) according to European LeukemiaNet recommendations [19]. Additional information on sequencing and flow cytometry to evaluate MRD are provided in the Supplementary Materials.

### 2.7. Quality of Life Assessments

Patients completed the EORTC QLQ-C30 and the QOL-E questionnaires at baseline, after first induction cycle, on the visit prior to randomization, at 2, 4, and 6 months after randomization, and every 6 months until the end of the trial. Each assessment was performed prior to any other assessment scheduled for the visit on the same day. 

### 2.8. Statistical Analysis Plan

The sample size at randomization was calculated according to the proportions of patients alive and disease-free at 2 and 5 years. These proportions, defining the alternative hypothesis, were expected to be 0.15 and 0.05 in the control group and 0.50 and 0.30 in the AZA arm. According to this model, 27 subjects were required in each arm to detect a difference between the survival curves after 2 and 5 years, with power of 0.80 and a two-sided level of significance of 0.05. We considered that 40% of the patients enrolled would reach the randomization time point, so that approximately 136 patients were required to be included in the study. 

Patient characteristics, including demographics, concomitant diseases, AML characteristics, and all other variables were collected by electronic CRF (Dielnet SrL, Reggio Calabria, Italy).

Data were summarized as mean and standard deviation, median and interquartile range (IQR), or absolute frequency and percentage, as appropriate. Between group comparisons of continuous variables were performed by independent *t*-test or Mann–Whitney U test whereas within-group comparisons were performed by paired *t*-test or Wilcoxon test, as appropriate. 

#### 2.8.1. Disease-Free Survival

Analysis of DFS was performed according to the allocation arm at predefined time points (2 and 5 years) by the Kaplan–Meier method and the two curves were compared using the log-rank test. The effect of the allocation arm on DFS at 2 and 5 years was further investigated by crude and cytogenetic risk-adjusted Cox regression analyses. Data were expressed as hazard ratios (HRs), 95% confidence intervals (CIs), and *p* values. The adjustment for cytogenetic risk (codified in binary terms: low/intermediate vs. high) was performed based on pathophysiological considerations and previous papers published in the field [14]. The efficacy of AZA vs. BSC on DFS at 2 and 5 years was also investigated by Kaplan–Meier analysis according to age categories (below/above the median age value) and MRD status. 

The effect of MRD on DFS at 2 and 5 years was investigated in the whole sample and separately by study arm. The potential modification by age (below/above the median age; 68 years), cytogenetic risk, MRD, and TP53 mutation on the effect of AZA vs. BSC on DFS at 2 and 5 years was investigated by including into the same Cox regression analysis each potential effect modifier, the allocation arm, and the interaction (multiplicative) term between each candidate effect modifier and the allocation arm. 

#### 2.8.2. Number of Hospitalizations

The effect of allocation arm on the frequency of hospitalizations was investigated by comparing (Fisher’s exact test) the proportion of patients who were hospitalized in the post-random phase in the two study groups. The sum of the days of hospitalization as well as the median number (and IQR) of days of hospitalization per patient in the post-random phase were also reported by study arms.

#### 2.8.3. Quality of Life

Standardized QoL scores were calculated according to the authors’ instructions for QOL-E and EORTC QLQ-C30 questionnaires. All scales had standardized scores ranging between 0 and 100. Higher scores indicate better QoL, except for EORTC-QLQ C30 symptom scales, and scales were scored if the patient answered at least half of the items in a multi-item scale.

A change in the value of scores from baseline and between arms can be investigated by minimal clinically importance difference (MCID) that represents the cut-off value to distinguish patients experiencing a significant change in PRO scores. If the PROM does not provide a pre-established MCID value, it was generated after all the patients have been included in the trial based on the baseline PRO score. A MCID was defined as ≥0.5 standard deviations of the baseline domain score (assessed at diagnosis) for all QOL-E and EORTC QLQ-C30 domains [20].

The effect of the allocation arm on PRO data as well as on the achievement of MCID was investigated by crude and age-adjusted linear mixed models (LMMs) and generalized estimating equations (GEEs), respectively. In LMMs, data were given as regression coefficients (expressing the magnitude of treatment effect), 95% CIs, and *p* values. In GEE models, data were expressed as OR, 95% CIs, and *p* values.

#### 2.8.4. Overall Survival

The analysis of OS was planned according to the allocation arm at predefined time points (2 and 5 years) by the Kaplan–Meier method for comparison between two curves by the log rank test and data expressed as HRs, 95% CIs, and *p* values. 

Data analysis was performed by two commercially available statistical software: SPSS for Windows Version 22, IBM, USA, and STATA 16 StataCorp, Lakeway Drive, College Station, TX, USA.

## 3. Results

### 3.1. Patients

A total of 149 patients were enrolled to randomize 54 patients. At study closure, 1 patient in BSC was still in the study (3 years and 10 months follow-up post randomization). The main characteristics of patients at diagnosis are described in Table 1.

### 3.2. Pre-Randomization Phase

**Figure 1:**
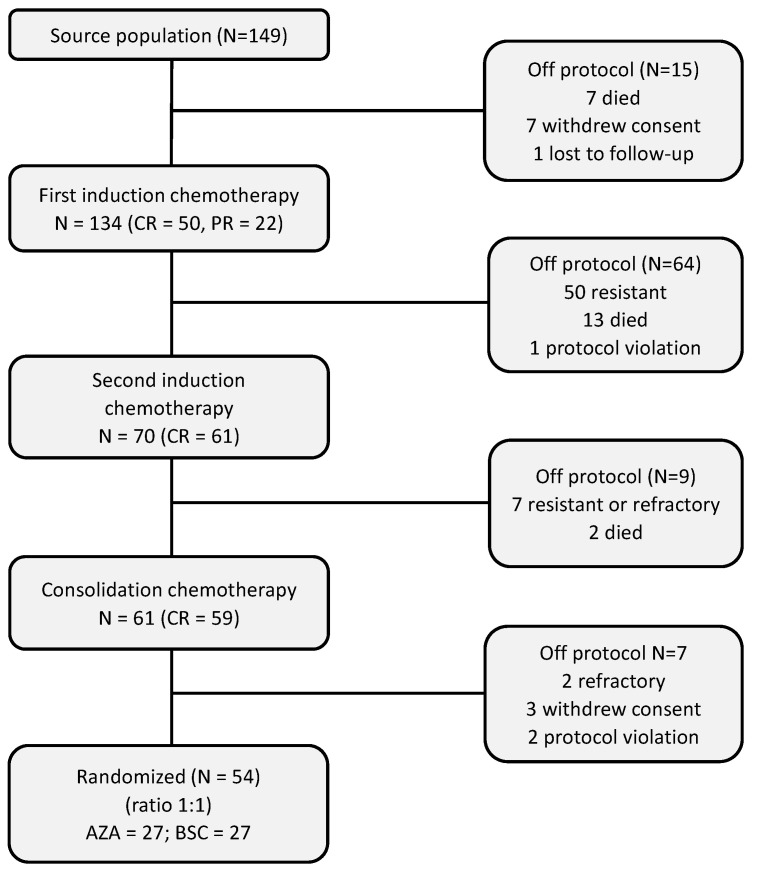
CONSORT flow diagram of pre-randomization phase. AZA = azacitidine, BSC = best supportive care, CR = complete remission, PR = partial remission.

Seven patients (4.7%) died before starting induction chemotherapy. After the first induction cycle, 50 patients (33.6%) obtained a CR and 22 (14.8%) a partial remission (PR), and 50 patients (33.6%) were resistant, while 13 (8.7%) died during first induction chemotherapy (causes of death are shown in the Supplementary Materials Table S1). Seven subjects refused to continue and one was lost on follow-up. A CONSORT flow diagram of the detailed pre-randomization phase is shown in Figure 1. A total of 54 patients were randomized (27 in AZA arm and 27 in BSC arm). 

### 3.3. Post-Randomization Phase

The main characteristics of the 54 patients at randomization and according to random arm are shown in Table 2. Age differed (*p* = 0.069) with a nearly 3-year trend difference in mean age between the two allocation arms (Supplementary Materials Figure S2).

Twenty-three patients had an increase in the dose of AZA after the 1st cycle, 3 patients did not increase the dose due to neutropenia, and 1 patient did not increase the dose due to being off-protocol after 1st cycle relapse.

### 3.4. Primary Endpoint: Disease-Free Survival

During the post-randomization phase, 43 (79.6%) subjects relapsed. At 2 years post randomization, no deaths occurred prior to relapse, thus OS was not assessed. Twenty-two patients in the BSC arm relapsed with a median DFS of 6.0 (95% CI: 0.2–11.7) months vs. 18 patients in the AZA arm, having a median DFS of 10.8 (95% CI: 1.9–19.6) months. 

**Figure 2:**
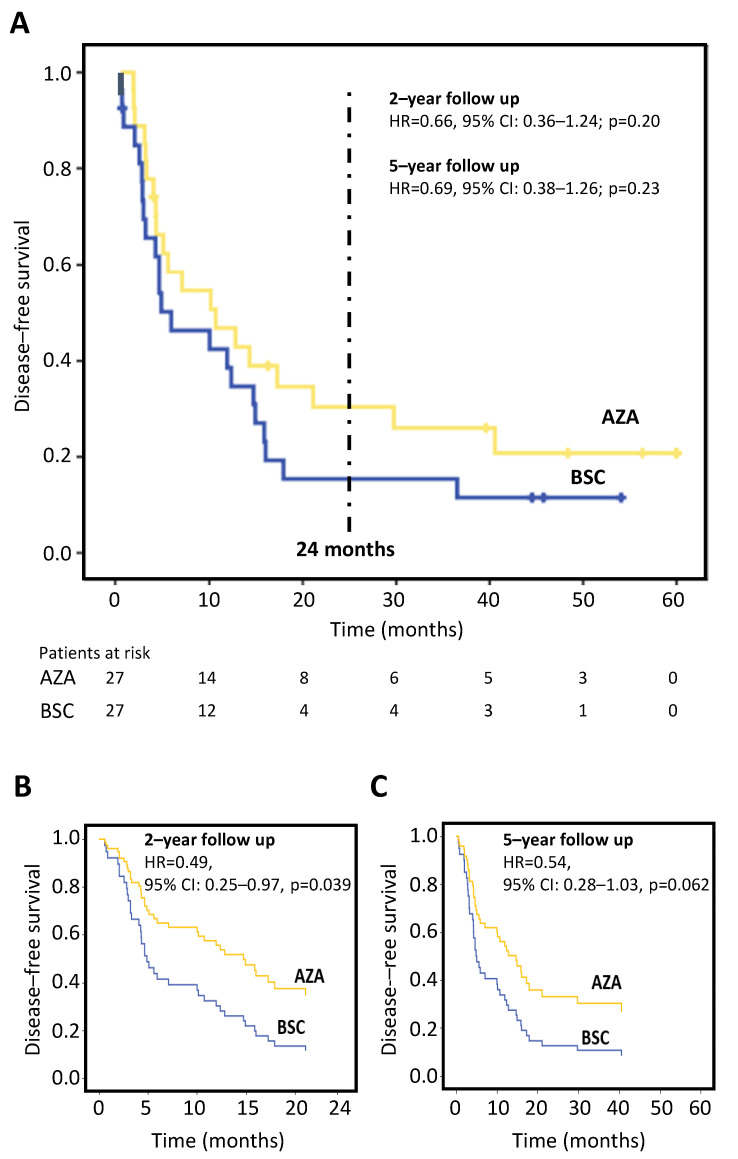
Kaplan–Meier analysis of disease-free survival. (**A**) Disease-free survival in all 54 randomized patients in AZA vs. BSC arms. Cox regression survival curves in which the allocation arm is adjusted for cytogenetic risk (low/intermediate vs. high) after a follow-up period of 2 years (**B**) and 5 years (**C**). This adjustment generates two expected survival curves based on two covariates in the model. AZA = azacitidine; BSC = best supportive care, CI = confidence interval, HR = hazard ratio.

At 5 years post randomization, none of the patients died before relapse; 2 patients on AZA and 1 patient on BSC withdrew consent and 1 patient on AZA withdrew for relapse of bladder cancer in CR. In the BSC arm, 23 patients relapsed with a median DFS of 6.0 (95% CI: 0.2–11.7) months vs. 20 patients in the AZA arm, with a median DFS of 10.8 (95% CI: 1.9–19.6) months. The differences did not reach statistical significance (Figure 2A). The cumulative relapse-free survival at 2 years was 30.3% (95% CI: 12.3–48.3) in AZA arm vs. 15.4% (95% CI: 1.48–29.3) in the BSC arm (*p* = 0.20), and at 5 years was 20.8% (95% CI: 4.1–37.5) in the AZA arm vs. 11.6% (95% CI: 0.0–23.9) in the BSC arm (*p* = 0.23).

After data adjustment for cytogenetic risk, the effect of AZA maintenance on DFS was statistically significant at 2 years in the AZA arm vs. BSC arm (hazard ratio; HR = 0.49, 95% CI: 0.25–0.97, *p* = 0.039) (Figure 2B) and just failed to attain statistical significance at 5 years (HR = 0.54, 95% CI: 0.28–1.03, *p* = 0.062) (Figure 2C).

**Figure 3:**
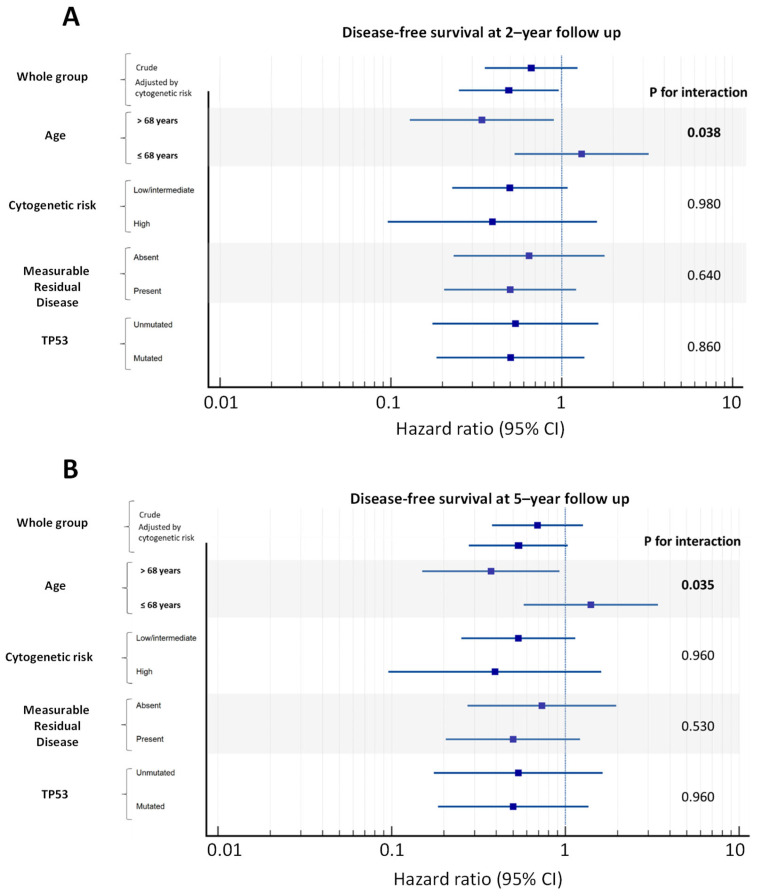
Hazard ratios of AZA vs. BSC for disease-free survival by relevant patients’ strata. (**A**) Disease-free survival by relevant patients’ strata at 2-year follow-up and (**B**), 5-year follow-up. Data presented as hazard ration and 95% CI. CI = confidence interval.

Although data adjustment for cytogenetic risk improved the allocation arm study outcome, cytogenetic risk per se did not significantly modify the effect of AZA vs. BSC on DFS both at 2 and 5 years of follow-up (Figure 3).

MRD was obtained by immunophenotype alone in 9 cases, by PCR alone in 10 cases, and by both measures in 28 cases (Table 2). Out of 40 patients who had MRD status, 25 were observed to have positive MRD status with a normal karyotype and this remained unchanged during the post-remission phase. In the whole study sample (i.e., independently of the allocation arm), the effect of MRD on DFS was found to be statistically significant both at 2 years (HR = 1.95, 95% CI: 1.01–3.82, *p* = 0.049) and 5 years post randomization (HR = 1.97, 95% CI: 1.03–3.78, *p* = 0.041). Data analysis by study arms revealed a statistically significant relationship between MRD and 2- and 5-year DFS (HR = 2.93, 95% CI: 1.06–8.05, *p* = 0.038 and HR = 2.93, 95% CI: 1.06–8.06, *p* = 0.038 respectively) in the control arm but not in the AZA arm (HR = 1.73, 95% CI: 0.66–4.55, *p* = 0.269 and HR = 1.69, 95% CI: 0.68–4.21, *p* = 0.260 respectively). 

Over the 5-year period, there was no difference between MRD-negative patients on treatment with AZA and on treatment with BSC (for both, the median DFS was 13 months). Instead, a difference between MRD-positive patients on treatment with AZA was observed (median DFS = 7 months) and on treatment with BSC (median DFS = 4 months) but this difference did not achieve statistical significance (log-rank test: 2.44, *p* = 0.118).

As age tended to differ between allocation arms, it was specifically tested as a potential confounder and effect modifier by stratifying the study population into two groups, i.e., below and above the median value of age of 68 years. As shown in Figure 3, age significantly modified the effect of AZA vs. BSC on DFS both at 2 and 5 years of follow-up (effect modification, *p* = 0.038 and *p* = 0.035). 

**Figure 4:**
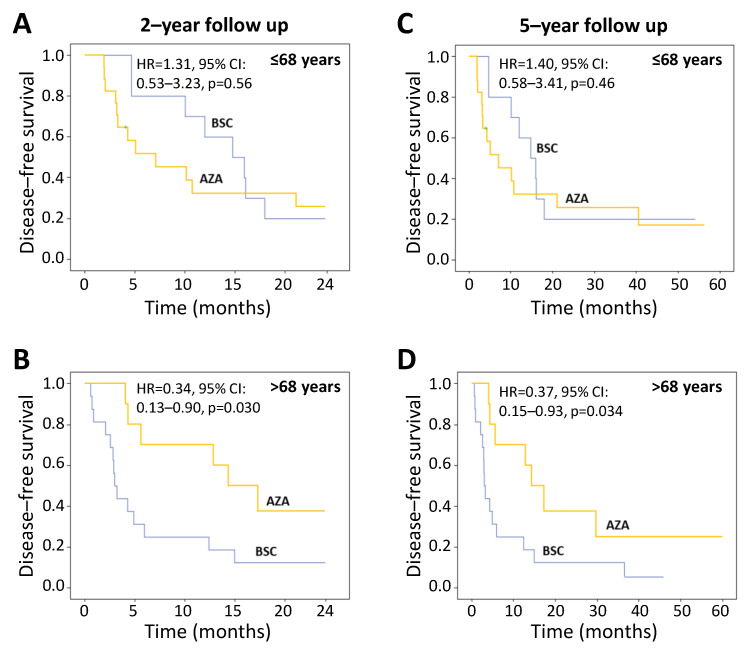
Kaplan–Meier analysis of disease-free survival by age strata. The hazard ratios were derived by Cox regression analyses. (**A**), disease-free survival up to 2 years in patients ≤68 years and (**B**), in patients >68 years. (**C**), disease-free survival at 5-year follow-up in patients ≤68 years and (**D**), in patients >68 years. AZA = azacitidine; BSC = best supportive care, CI = confidence interval, HR = hazard ratio.

In fact, while no difference was reported on DFS between AZA and BSC arms in patients aged ≤68 years, DFS at 2 and 5 years in patients aged >68 years was significantly longer in the AZA arm (HR 0.34, 95% CI: 0.13–0.90, *p* = 0.030 and HR 0.37, 95% CI: 0.15–0.93, *p* = 0.034, respectively) (Figure 4). Of note, data adjustment for cytogenetic risk further amplified the efficacy of AZA in patients aged >68 years, both over a 2-year (HR 0.24, 95% CI: 0.08–0.69, *p* = 0.008) and 5-year period (HR 0.28, 95% CI: 0.10–0.76, *p* = 0.012).

At diagnosis, TP53 mutational status was available in 40 patients. In 19 (47.5%) cases, TP53 was mutated, and 9 subjects lost the mutation after induction chemotherapy. Independently of the allocation arm, the effect of TP53 at diagnosis on DFS resulted to be statistically significant both at 2 years (HR 2.43, 95% CI: 1.17–5.07, *p* = 0.018) and 5 years post randomization (HR 2.62, 95% CI 1.27–5.42, *p* = 0.009). Furthermore, TP53 did not modify the effect of AZA vs. BSC on DFS at 2 and 5 years of follow-up (Figure 3).

Of the 10 patients in the AZA arm aged >68 years, their characteristics at baseline are as follows: 1 patient had missing karyotype, 7 patients had a normal karyotype, 1 patient had a complex karyotype and 1 patient had del(3p). Five patients harbored a TP53 mutation. Mean Hb levels were 9.1 g/dL (±1.2 g/dL), mean white blood cell count was 11.3 × 10^3^ (±16.9 × 10^3^), mean platelet count was 35.4 × 10^3^ (±25.5 × 10^3^), and median bone marrow blast count was 71.5% (IQR 60.0–82.5%). Five patients reached randomization with MRD positivity and four with mutant TP53.

### 3.5. Safety

During the post-randomization phase, 75 treatment-emergent adverse events occurred and were significantly (*p* = 0.007) more frequent in the AZA arm (N = 60) than in the BSC arm (N = 15) (Supplementary Materials Figure S3). Significantly more subjects (N = 17) in the AZA arm had at least one adverse event vs. 9 subjects in best supportive care. In particular, 21 grade 3–4 treatment-emergent adverse events occurred, 20 in AZA arm and 1 in BSC arm (*p* = 0.002; Table 3). Neutropenia was the most frequently observed adverse event. Bladder cancer occurred in one case. 

### 3.6. Secondary Endpoints

#### 3.6.1. Number and Duration of Hospitalizations during the Study

In the AZA arm, 2 patients out of 27 (7.4%) were hospitalized whereas no patient was hospitalized in the BSC arm. Reasons for hospitalization were AEs in 2 patients (1 with bladder cancer and 1 suspected pericarditis with abdominal pain). 

#### 3.6.2. Quality of Life

QoL and symptom (patient-reported outcomes, PROs) scores of all available patients (n = 111) are illustrated in Supplementary Materials Table S2. Scores were generally poor, though fatigue was not a prevalent issue. The highest impact was revealed in functional scores.

Following induction therapy, there was a significant improvement in PRO measures in patients achieving a CR in almost all domains except EORTC QLQ-C30 role and social function (Supplementary Materials Table S3). As shown in Supplementary Table S4, the majority of the scores of the PRO domain measures remained stable or improved whereas only a minority worsened. The mean and median changes of QOL-E and EORTC QLQ-C30 domains and relative MCIDs are shown in Supplementary Materials Table S5.

The effect of AZA vs. BSC on the changes of PRO scores over time, in particular on the achievement of MCID for each domain, is reported in Supplementary Materials Tables S6 and S7. There was no significant impact of maintenance therapy on patient-reported outcomes and there were no differences in PROs between arms. A between-arm comparison of the mean and median changes of each QoL domain and the proportions of patients who remained stable, improved, or worsened according to the MCID showed no clinically meaningful differences during the post-randomization phase (see Supplementary Materials Tables S8 and S9). 

## 4. Discussion

Maintenance treatment is at present employed in many hematologic malignancies to prolong CR by reducing the risk of disease relapse [21,22]. However, the role of maintenance during the post-remission phase of patients with AML is still a matter of debate and many previous studies either with conventional low-dose chemotherapies, or targeted biologic treatments, failed to demonstrate its efficacy in prolonging disease-free survival [23,24]. 

AZA in biologic and clinical profiles make its use very attractive as maintenance therapy, due to its relatively low toxicity and its efficacy; in particular, elderly patients could benefit by this maintenance approach. Notwithstanding the above considerations, however, only a few studies have been conducted employing AZA as maintenance of CR in AML patients.

Initially, three non-randomized phase-2 trials reported on the use of low-dose AZA (50 mg/m^2^ for 5 days), confirming both feasibility and low rate of adverse events in small cohorts of elderly patients [25,26,27]. Maintenance with hypomethylating agents, including AZA, was also tested after allogenic/autologous transplantation: data from these studies have been recently summarized [28] and showed favorable results on survival and graft versus host disease.

More recently, two larger randomized phase-3 trials on AZA maintenance vs. placebo/best supportive care in elderly AML patients were conducted and published. In the HOVON trial [14], similar to the one in the present report, subcutaneous AZA was investigated. However, three major differences should be highlighted: (1) intensive induction treatment was at a physician’s judgement and patients were included in the study after two induction cycles without consolidation phase, (2) AZA dosage was fixed and lower than in our trial (50 mg/m^2^ for 5 days), and (3) maintenance had a fixed 12-month duration. The QUAZAR AML-001 trial [15] investigated the efficacy and safety of the oral formulation of AZA. Again, in that trial, the induction phase was at the discretion of the treating physician, patients aged >55 (younger) were enrolled and randomized upon achieving a CR, and patients with initial relapse during maintenance (marrow blasts <15%) could be rescued with increments of AZA dosing (21 days instead of 14 days every 28 days). Considering the aforementioned (non-trivial) differences, DFS in the AZA arm was significantly longer in these two trials as well as in the present study, highlighting the efficacy of the active AZA treatment in delaying relapse occurrence. 

Among several factors evaluated in our cohort for their possible impact on efficacy of AZA maintenance compared to placebo, only those of older age (>68 years) emerged as being significantly associated with a better outcome. This finding is in line with data from a previous phase-2 study, in which decitabine maintenance provided no benefit in patients aged <60 years [29]. From a speculative point of view, it is somewhat expected because AZA has shown greater efficacy in marrows with myelodysplastic features and the rate of AML evolving from a previous myelodysplastic phase increases with age. Furthermore, MRD and TP53 mutation did not impact the efficacy of AZA in our cohort.

It also important to emphasize that MRD positivity was observed to have a negative effect on DFS in BSC but not in patients receiving AZA, which supports the use of AZA maintenance especially for MRD-positive cases, a finding that was not observed in exploratory analyses of the QUAZAR AML-001 trial where AZA was shown to prolong survival in patients with AML in remission independently of MRD status [30].

Our study reported a good feasibility of AZA maintenance, with neutropenia representing the most common toxicity, accounting for the majority of grade 3–4 adverse events in the AZA arm and with the absence of mortality prior to progression. It is worth noting that neutropenia was also reported as an important adverse event in the QUAZAR AML-001 trial; however, in gastrointestinal toxicity due to the oral formulation of AZA also represented an adverse event, which is virtually absent with subcutaneous infusion. 

Moreover, in our study, the expected higher rate of adverse events in the AZA arm and the burden of receiving treatment did not translate into worse patient-reported outcomes (PRO) compared to the placebo arm. As already reported [31], PRO scores in comparison with those at diagnosis significantly improved in patients achieving CR as during the maintenance phase, no difference was observed between the two arms, underscoring the lack of negative impact by AZA treatment in all PROs.

Patients in the present study were elderly (median age of 69) and when this trial was undertaken in 2010, allogenic stem cell transplantation was not the mainstay therapy offered. Currently, this approach is considered the mainstay treatment for younger AML patients with intermediate or high-risk MDS and offers superior efficacy compared to non-allograft stem cell approaches [32,33]. Despite this, approximately one third of patients still suffer from disease relapse, leading to poor outcomes [34,35]. There is also evidence that the use of hypomethylating agents, such as AZA, as post-remission therapy after allograft stem cell transplantation were shown to have an improved outcome [28]. 

Indeed, the use of the allograft transplant approach has significantly improved in the past decade [36]; however, in elderly patients (≥60 years), the decision to offer allograft stem cell transplantation still remains a topic of debate in this population, since the toxicity of conditioning regimens, the risk of graft-versus-host disease, and the need for prolonged immunosuppression are major concerns for these vulnerable patients [37,38]. Furthermore, from a practical point of view, the feasibility can be challenging due to lack of familial donor availability (HLA identical sibling donor) and given their advanced age. 

It should be emphasized that patients in this trial underwent very intensive treatment (two inductions + high dose consolidations), which are normally not administered to this patient population, unless the intent is to pursue stem cell transplantation that could diminish the impact of AZA maintenance of DFS and OS. Nevertheless, our results argue that the benefit of post-remission therapy may be limited to older patients who cannot tolerate or who cannot complete the full course of intensive induction/consolidation.

## 5. Study Limitations

This trial was designed before 2010 considering previous 2003 response criteria [16]. Although allogenic transplantation was being offered more frequently in subsequent years, particularly in the USA [39], this treatment was not standard treatment in Italy for the age group under our study. Indeed, in economically disadvantaged countries or where allogenic transplantation cannot be offered, results from our trial provide additional treatment options in elderly AML patients. We did not follow up on survival after relapse, losing potentially important information on AZA maintenance treatment. We used delayed cycling (56-day cycle) that may have impacted on reduced efficacy. A higher number of patients may have contributed to a more robust analysis.

## 6. Conclusions

This trial evaluated the efficacy and feasibility of AZA maintenance in elderly AML patients and particular benefit was observed in subjects aged >68 years. The most effective schedule and dosing still remain a matter of debate and may be individualized. 

## Figures and Tables

**Table 1:** Baseline characteristics of patients.

Characteristics	All Patients (N = 149)
Age, median years (IQR)	69 (65–74)
Male, N (%)	78 (52%)
AML de novo, N (%)	121 (81%)
Hemoglobin, mean g/dL (±SD)	9.1 ± 1.4
White blood cell × 10^3^, median (IQR)	7.9 (2.5–28.8)
Platelet × 10^3^, median (IQR)	52 (26–84)
WHO Classification, N (%)	
*AML with minimal differentiation*	28 (18.8%)
*Acute myelomonocytic leukemia*	28 (18.8%)
*AML with myelodysplasia-related changes*	28 (18.8%)
*AML with maturation*	24 (16.1%)
*Acute monoblastic and monocytic leukemia*	17 (11.4%)
*AML without maturation*	12 (8.1%)
*AML with recurrent genetic abnormalities*	8 (5.3%)
*Therapy-related myeloid neoplasms*	2 (1.3%)
*Acute erythroid leukemia*	1 (0.7%)
*Acute megakaryoblastic leukemia*	1 (0.7%)
Cytogenetic risk profile, N (%)	
*Good*	1 (0.7%)
*Intermediate*	104 (69.8%)
*Poor*	26 (17.4%)
*Not evaluable*	18 (12.1%)

AML = acute myeloid leukemia, WHO = World Health Organization.

**Table 2:** Characteristics of patients at randomization.

Characteristics	AZA (N = 27)	BSC (N = 27)	All Patients (N = 54)	*p* Value
Age, mean years (±SD)	67.7 ± 5.2	70.4 ± 5.5	69.1 ± 5.5	0.069
Male, N (%)	17 (63%)	14 (52%)	78 (57%)	0.583
AML de novo, N (%)	21 (78%)	26 (96%)	47 (87%)	0.100
Hemoglobin, mean g/dL (±SD)	8.9 ± 1.0	9.3 ± 1.3	9.1 ± 1.2	0.206
White blood cell × 10^3^, median (IQR)	4.1 (2.1–23.8)	17.0 (2.5–25.7)	6.0 (2.2–24.4)	0.382
Platelet × 10^3^, median (IQR)	39 (26–63)	54 (24–77)	42 (26–74)	0.574
BM blasts (%), median (IQR)	70 (49–80)	70 (50–90)	70 (50–89)	0.696
PB blasts (%), median (IQR)	17 (3–70)	40 (15–75)	32 (8–70)	0.158
WHO Classification, N (%)				0.259
*AML with minimal differentiation*	6 (22%)	3 (11%)	9 (17%)	
*Acute myelomonocytic leukemia*	4 (15%)	7 (26%)	11 (20%)	
*AML with myelodysplasia-related changes*	6 (22%)	1 (4%)	7 (13%)	
*AML with maturation*	3 (11%)	6 (22%)	9 (17%)	
*Acute monoblastic and monocytic leukemia*	3 (11%)	5 (19%)	8 (15%)	
*AML without maturation*	1 (4%)	1 (4%)	2 (4%)	
*AML with recurrent genetic abnormalities*	3 (11%)	2 (7%)	5 (9%)	
*Therapy-related myeloid neoplasms*	0	2 (7%)	2 (4%)	
*Acute erythroid leukemia*	1 (4%)	0	1 (2%)	
Baseline cytogenetic risk profile, N (%)				0.375
*Good*	1 (4%)		1 (2%)	
*Intermediate*	19 (70%)	22 (81%)	41 (76%)	
*Poor*	4 (15%)	4 (15%)	8 (15%)	
*Not evaluable*	3 (11%)	1 (4%)	4 (7%)	
MRD at random, N (%)				0.861
*Present*	13 (48.1%)	12 (44.4%)	25 (46.3%)	
*Absent*	12 (44.4%)	10 (37.0%)	22 (40.7%)	
*Not evaluable*	2 (7.5%)	5 (18.6%)	7(13.0%)	
Mutation at random, N (%)				
*FLT3*	2 (7.4%)	1 (3.7%)	3 (5.6%)	0.315
*NPM1*	1 (3.7%)	4 (14.8%)	5 (9.3%)	0.343
*IDH1*	2 (7.4%)	0	2 (3.7%)	0.232
*IDH2*	1 (3.7%)	3 (11.1%)	4 (7.4%)	0.606
TP53 at diagnosis	10 (37.0%)	9 (33.3%)	19 (35.2%)	0.752

AML = acute myeloid leukemia, AZA = azacitidine, BSC = best supportive care, IQR = interquartile range, MRD = measurable residual disease, SD = standard deviation, WHO = World Health Organization.

**Table 3:** Grade III/IV adverse events.

Adverse Event	AZA		BSC	
	Grade 3	Grade 4	Grade 3	Grade 4
Neutropenia	5	5	1	0
Thrombocytopenia	3	0	0	0
Anemia	2	0	0	0
Pneumonia	1	0	0	0
CVC bacterial infection	1	0	0	0
Pericarditis	1	0	0	0
Abdominal pain	0	1	0	0
Urothelial bladder cancer	1	0	0	0
Total	14	6	1	0

AZA = azacitidine, BSC = best supportive care, CVC = central venous catheter.

## Data Availability

Supplementary Materials accompanies this paper. Raw data are available upon request from the corresponding author (E.N.O.).

## Supplementary Materials

The following supporting information can be downloaded at: https://www.mdpi.com/article/10.3390/cancers15092441/s1, References [19,40] are cited in the supplementary materials. Principal investigators and study sites, Supplementary Methods, Supplementary Figure S1, Trial design, Supplementary Results; Supplementary Table S1, Causes of death before randomization; Supplementary Figure S2, Distribution of cases by random allocation and by age; Supplementary Figure S3, Treatment-emergent adverse events; Supplementary Table S2, QOL-E and EORTC QLQ-C30 scores at baseline (diagnosis) (n = 111) Supplementary Table S3, QOL-E and EORTC QLQ-C30 scores from diagnosis of AML to randomization; Supplementary Table S4, The proportions of patients who remained stable, improved, or worsened according to the MCID from diagnosis to randomization; Supplementary Table S5, Mean and median changes in QOL-E and EORTC QLQ-C30 domains from diagnosis to randomization; Supplementary Table S6, Linear mixed model analyses of PRO measures quality of life after randomization; Supplementary Table S7, Generalized estimating equation of PRO measures after randomization; Supplementary Table S8, The MCIDs and changes in PRO scores in the first 6 months following randomization according to allocation arm; Supplementary Table S9, The proportions of patients who remained stable, improved, or worsened according to the MCID after randomization.
