# Supplementary material for: Azacitidine Post-Remission Therapy for Elderly Patients with AML: A Randomized Phase-3 Trial (QoLESS AZA-AMLE)
Source: Cancers (Basel). 2023 Apr 24;15(9):2441. doi: 10.3390/cancers15092441 (PMC10177242; doi:10.3390/cancers15092441)
Supplement: Supplementary file 1 [file cancers-15-02441-s001.zip › cancers-2286226-supplementary.pdf]

Azacitidine post-remission therapy for elderly patients with AML: a randomized phase-2 trial (QoLESS AZA-AMLE)

SUPPLEMENTARY MATERIAL

Principal Investigators and Study Sites.. 2

SUPPLEMENTARY METHODS.. 3-6

Figure S1. Trial design……………………………………...……. 3

Mutation sequencing and flow cytometry…………………………………………. 4-6

SUPPLEMENTARY RESULTS 7-22

Table S1. Causes of death before randomization....................... 7

Figure S2. Distribution of cases by random allocation and by age...8

Figure S3. Treatment-emergent adverse events........................ 9

Table S2. QOL-E and EORTC QLQ-C30 scores at baseline (diagnosis) (N=111)... 10

Table S3. QOL-E and EORTC QLQ-C30 scores from diagnosis of AML to randomization...11

Table S4. The proportions of patients who remained stable, improved, or worsened according to the MCID from diagnosis to randomization... 12

Table S5. Mean and median changes in QOL-E and EORTC QLQ-C30 domains from diagnosis to randomization…... 13-14

Table S6. Linear mixed model analyses of PRO measures quality of life after randomization………………... 15

Table S7. Generalised estimating equation of PRO measures after randomization……………………………………………..........................16

Table S8. The MCIDs and changes in PRO scores in the first 6 months following randomization according to allocation arm…………………… 17-20

Table S9. The proportions of patients who remained stable, improved, or worsened according to the MCID after randomization……………………………. 21-22

Principal investigators and study sites

Investigator	Center (study site)	Italian city/town	Enrolled subjects	Randomized subjects
Esther Oliva	Grande Ospedale Metropolitano Bianchi Melacrino Morelli	Reggio Calabria	18	7
Anna Candoni	P.O. Santa Maria Della Misericordia, A.S.U.F.C Di Udine	Udine	31	12
Prassede Salutari	Ospedale Civile Spirito Santo Pescara	Pescara	25	9
Francesco Di Raimondo	AOU “Policlinico-V.Emanuele” Catania	Catania Ferrarotto	17	9
Gianluigi Reda	Università Degli Studi Di Milano, IRCCS Ospedale Maggiore Policlinico Milano	Milano Policlinico	15	5
Deborah Capelli	Azienda Ospedaliera Universitaria - Ospedali Riuniti Di Ancona	Ancona	12	4
Pasquale Niscola	Ospedale Sant'eugenio Roma	Roma Sant’Eugenio	8	2
Carmine Selleri	Aou San Giovanni Di Dio E Ruggi D'aragona, Salerno	Salerno	7	2
Pellegrino Musto	Centro Di Riferimento Oncologico Di Basilicata Rionero In Vulture	Rionero in Vulture	4	2
Ernesto Vigna	Ospedale L'annunziata, Cosenza	Cosenza	2	1
Antonio Volpe	Azienda Ospedaliera San Giuseppe Moscato, Avellino	Avellino	5	1
Nicola Cascavilla	Irccs Casa Sollievo Della Sofferenza	San Giovanni Rotondo	4	0
Donato Mannina	Azienda Ospedaliera Papardo, Messina	Messina	1	0
		**Total**	**149**	**54**

SUPPLEMENTARY METHODS

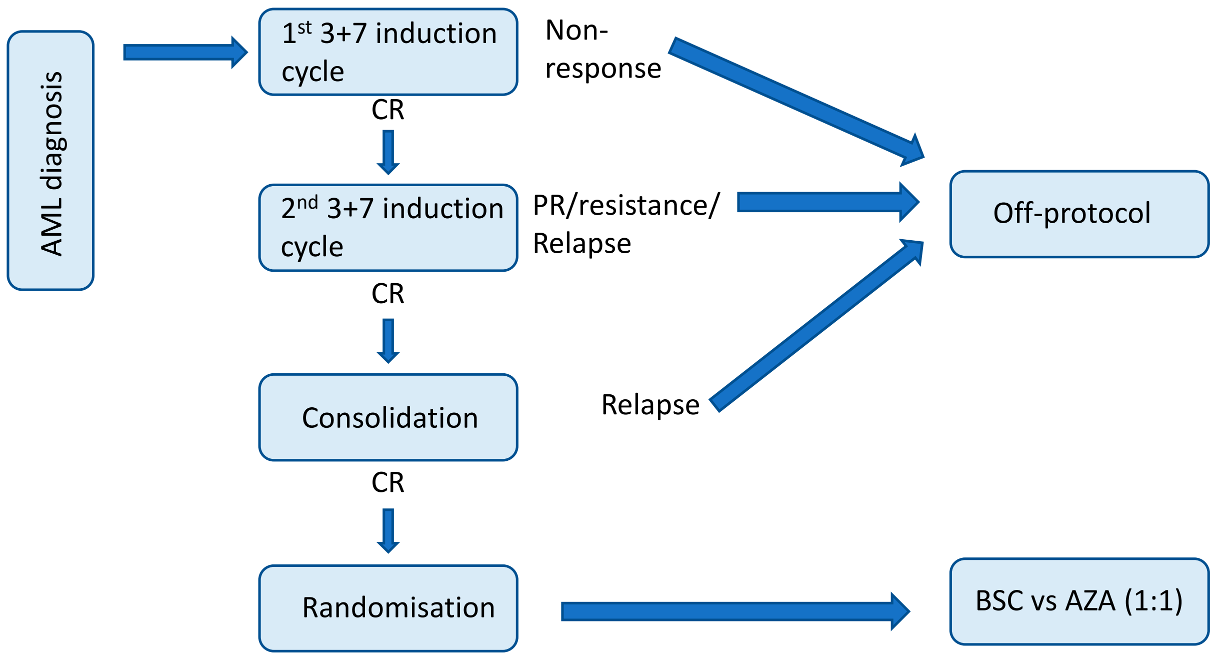

Figure S1. Trial design

MUTATION SEQUENCING

NPM1 analysis

The DNA was extracted according to standard protocol and 5uL of the diluted DNA was used to detect NPM1 mutation in PCR real Tieme. The Ipsogen (Qiagen) *NPM1* MutaScreen Kit detects total *NPM1* (wild-type + mutated) and mutated *NPM1* and separately identifies *NPM1* Mut A, Mut B, and Mut D in genomic DNA. Quantitative, real-time PCR was conducted using the NPM1 MutaQuant Assay A, B&D (Ipsogen, Qiagen) that includes primers and probes for NPM1 mutation and ABL, as well as NPM1 mutation and ABL cDNA samples for standard curve generation. Quantitative PCR was performed in a Quantstudio3 (ThermoFisher Scientific) with TaqMan Universal PCR Master Mix (Life Technologies).

FLT3-ITD analysis

For fragment analysis, PCR (amplification for 28 cycles (30 sec 94°C, 1 min 60°C, 1 min 72°C) with 1 µL patient gDNA (10 ng/µL) was performed in a 25 µL reaction volume to amplify FLT3, utilizing fluorescently-labelled primers (10 pmol each, ThermoFisher Scientific) as published [1] and the Taq PCR Master Mix (Qiagen). Thereafter, 0.5 µL fragment-length standard (GeneScan (500) ROX Size Standard (ThermoFisher Scientific) and 13.5 µL PCR-grade water were added to 1 µL PCR product. After initial denaturation of this mixture at 95°C, size-separation by capillary electrophoresis was performed on a Seqstudio Genetic Anayzer System (ThermoFisher Scientific). Data analysis was performed using the Gene-Mapper 6 software (ThermoFisher Scientific). FLT3-ITD mutational burden was calculated based on the WT to ITD ratio equation as previously published [1]

IDH1, IDH2 and TP53 sequencing

Mutational status was investigated using a custom panel Ion AmpliSeq (ThermoFisher Scientific) covering 100% of exonic positions and ±10 bp exon-intron boundaries. Briefly, libraries were automatically prepared on the Ion Chef System (ThermoFisher Scientific) using 10 ng of gDNA and Ion Chef for DL8 kit (ThermoFisher), following the manufacturer's instructions. Libraries were diluted to 10 pmol and multiplexed for template preparation and Ion 530 chip loading steps. Sequencing runs were performed on Ion S5 Genestudio (ThermoFisher), and a first analysis locally conducted on Torrent Suite Software (TSS, version 5.14, ThermoFisher) through a predefined

bioinformatics pipeline. Coverage and variant calling metrics were determined by running Coverage Analysis and Torrent Variant Caller (TVC) plugins. Variants annotation and filtering were performed by processing Variant Call Format (VCF) files on the Ion Reporter tool (version 5.14, Annotate variants workflow, ThermoFisher). VCF files were reviewed on Integrative Genomic Viewer software (IGV, version 2.7, Broad Institute). We established a cut-off of 3% as the minimum VAF to consider a mutation. The minimum coverage was 300x amplicon with optimal expected mean depth values of at least 1000x. HGVS nomenclature was used to describe the identified mutations. The synonymous and intronic variants, except those occurring at splice sites, were not considered in our analyses, as recommended by the ERIC consortium. The possible germline origin of those TP53 variants with at least 50% VAF was verified through non-leukemic cells TP53 testing (buccal swab) and consequently excluded from the analysis.

Flow cytometry

Multi-parameter multiparameter (commonly at least 3- to 4-color) flow cytometry was used to determine lineage involvement of a newly diagnosed acute leukemia according to European LeukemiaNet recommendations [2]. In brief, for most markers, a commonly used criterion is 20% or more of leukemic cells expressing the marker,10 whereas for selected markers (eg, cytoplasmic CD3, MPO, TdT, CD34, CD117) a lower cut-off has been applied (10%). Quantification of expression patterns of several surface and cytoplasmic antigens is necessary for lineage assignment, to diagnose mixed phenotype acute leukemia, and to detect aberrant immunophenotypes allowing for measurement of minimal residual disease (MRD).

1. N I Noguera , E Ammatuna, D Zangrilli, S Lavorgna, M Divona, F Buccisano, S Amadori, C Mecucci, B Falini, F Lo-Coco . Simultaneous detection of NPM1 and FLT3-ITD mutations by capillary electrophoresis in acute myeloid leukemia. Leukemia 2005 Aug;19(8):1479-82.

2. Döhner H, Estey EH, Amadori S, Appelbaum FR, Büchner T, Burnett AK, Dombret H, Fenaux P, Grimwade D, Larson RA, Lo-Coco F, Naoe T, Niederwieser D, Ossenkoppele GJ, Sanz MA, Sierra J, Tallman MS, Löwenberg B, Bloomfield CD; European LeukemiaNet. Diagnosis and management of acute myeloid leukemia in adults: recommendations from an

international expert panel, on behalf of the European LeukemiaNet. Blood. 2010 Jan 21;115(3):453-74.

SUPPLEMENTARY RESULTS

Table S1. Causes of death before randomization (N=22)

Event	Before induction, N	After first induction, N	After second induction, N
Infection	2	9	2
Heart failure	2	0	0
Bleeding	0	2	0
AML progression	1	1	0
Pulmonary embolism	1	0	0
Lung disease	1	0	0
Multiorgan failure	0	1	0

Figure S2. Distribution of cases by random allocation and by age

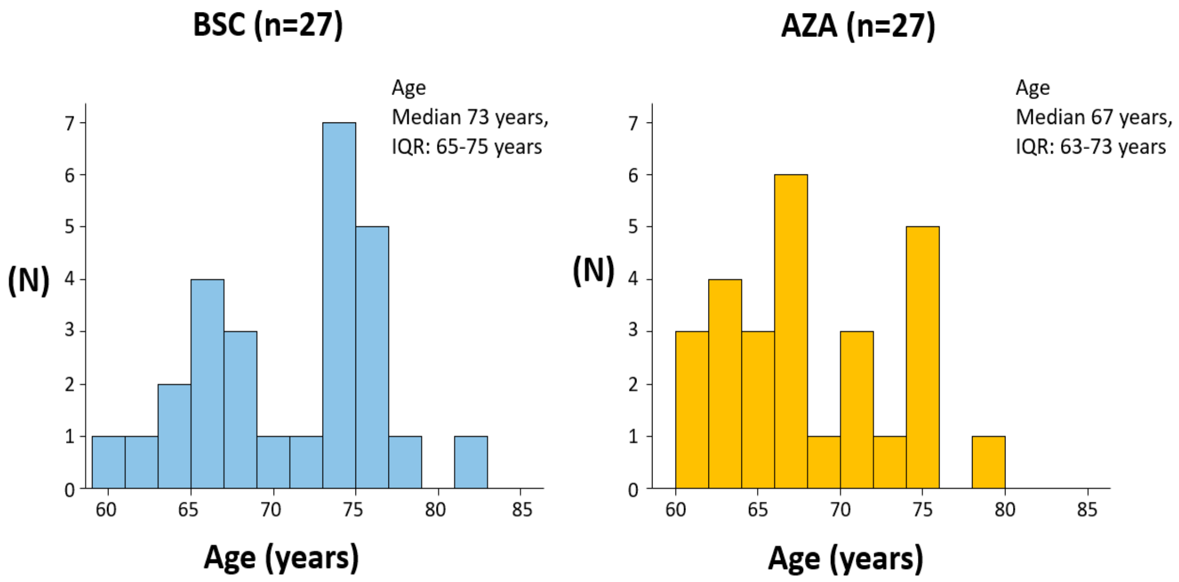

Figure S3. Treatment-emergent adverse events

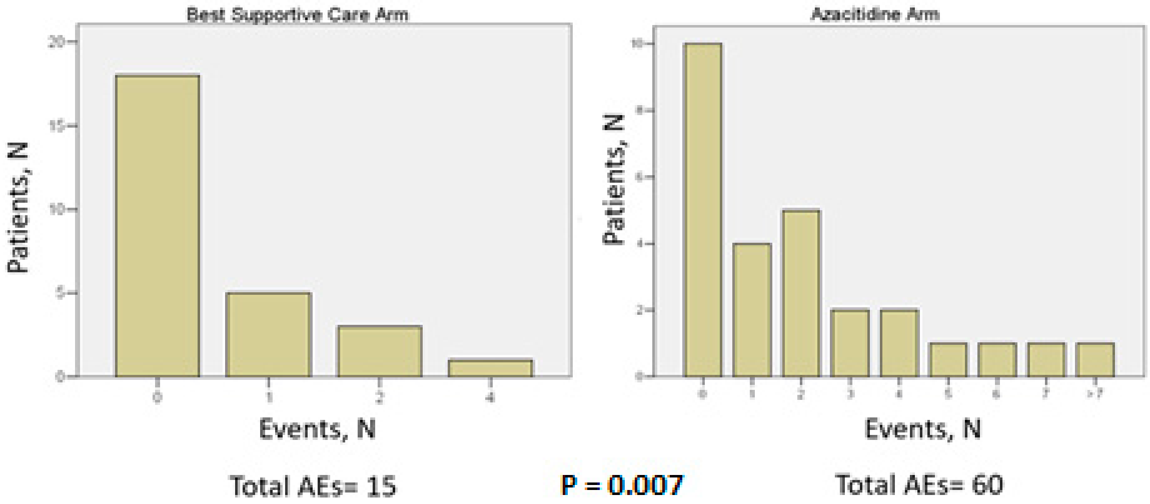

Table S2. QOL-E and EORTC QLQ-C30 scores at baseline (diagnosis) (N=111)

Domain	Baseline score Median (IQR)
QOL-E	
Physical	50 (37-69)
Functional	33 (22-75)
Social	50 (25-75)
Sexual	50 (25-100)
Fatigue	76 (52-86)
Disease specific	60 (33-74)
General	58 (42-72)
All	58 (44-70)
EORTC QLQ-C30	
Global	50 (33-67)
Physical	80 (60-87)
Social	67 (50-100)
Cognitive	83 (67-100)
Emotional	83 (58-92)
Role function	67 (50-83)
Fatigue	33 (22-56)
Nausea/vomiting	0 (0-17)
Pain	0 (0-33)
Dyspnoea	33 (0-33)
Insomnia	33 (0-33)
Loss of appetite	0 (0-33)
Constipation	0 (0-33)
Diarrhoea	0 (0-33)

IQR = interquartile range

Table S3. QOL-E and EORTC QLQ-C30 scores from diagnosis of AML to randomization

Domain	Baseline score		Post consolidation score		P-value
	N	Median (IQR)	N	Median (IQR)	
QOL-E					
Physical	39	63 (50-75)	39	63 (50-100)	0.014
Functional	37	33 (33-94)	37	89 (33-100)	0.028
Social	35	50 (25-75)	35	75 (50-100)	0.010
Sexual	14	67 (25-87)	14	100 (56-100)	0.016
Fatigue	40	79 (67-85)	40	86 (76-95)	<0.001
Disease specific	28	60 (36-85)	28	81 (71-93)	<0.001
General	11	64 (53-81)	11	79 (62-87)	0.110
Treatment outcome	22	60 (46-83)	22	82 (65-92)	0.005
All	9	68 (46 -82)	9	78 (66-93)	0.173
EORTC QLQ-C30					
Physical function	41	80 (60-87)	41	87 (70-97)	0.08
Role function	39	83 (50-100)	39	83 (67-100)	0.16
Social	39	67 (50-100)	39	83 (67-100)	0.14
Emotional	40	83 (67-92)	40	92 (83-100)	<0.001
Global health status	42	50 (33-75)	42	83 (65-83)	<0.001
Fatigue	41	33 (22-44)	41	11 (0-33)	0.002
Cognitive function	41	100 (83 – 100)	41	100 (83-100)	0.203
Nausea/vomiting	42	0 (0-0)	42	0 (0-16)	0.091
Pain	39	0 (0-33)	39	0 (0-16)	0.106
Dyspnoea	41	33 (0-50)	41	0 (0-33)	0.001
Insomnia	41	33 (0-33)	41	0 (0-17)	0.004
Appetite loss	42	0 (0-33)	42	0 (0-0)	0.016
Constipation	42	0 (0-33)	42	0 (0-33)	0.827
Diarrhoea	41	0 (0-33)	41	0 (0-0)	0.026
Financial problems	39	0 (0-0)	39	0 (0-33)	0.617

IQR = interquartile range

Table S4. The proportions of patients who remained stable, improved, or worsened according to the MCID from diagnosis to randomization

Domain	First induction				After consolidation			
QOL-E	N	Stable	Improved	Worsened	N	Stable	Improved	Worsened
Physical (%)	59	17 (28.8)	20 (33.9)	22 (37.3)	39	7 (17.9)	22 (56.4)	10 (25.6)
Functional (%)	52	28 (53.8)	13 (25.0)	11 (21.2)	37	16 (43.2)	16 (43.2)	5 (13.5)
Social (%)	51	25 (49.0)	12 (23.5)	14 (27.5)	35	14 (40.0)	17 (48.6)	4 (11.4)
Sexual (%)	28	17 (60.7)	8 (28.6)	3 (10.7)	14	7 50.0)	6 (42.9)	1 (7.0)
Fatigue (%)	57	32 (56.1)	12 (21.1)	13 (22.8)	40	21 (52.5)	16 (40.0)	3 (7.5)
Disease specific (%)	42	21 (50.0)	13 (31.0)	8 (19.0)	28	12 (42.9)	15 (53.6)	1 (3.6)
General (%)	14	8 (57.1)	3 (21.4)	3 (21.4)	11	4 (36.4)	6 (54.5)	1 (9.1)
All (%)	13	6 (46.2)	3 (23.1)	4 (30.8)	9	4 (44.4)	4 (44.4)	1 (11.1)
TOI (%)	34	16 (47.1)	9 (26.5)	9 (26.5)	22	7 (31.8)	13 (59.1)	2 (9.1)
EORTC QLQ-C30								
Global Health Status (%)	63	24 (38.1)	27 (42.9)	7 (19.0)	42	11 (26.2)	26 (61.9)	5 (11.9)
Physical function (%)	58	25 (43.1)	8 (13.8)	25 (43.1)	41	13 (31.7)	18 (43.9)	10 (24.4)
Role function (%)	63	25 (39.7)	18 (28.6)	20 (31.7)	39	9 (23.1)	18 (46.2)	12 (30.8)
Emotional function (%)	59	36 (61.0)	14 (23.7)	9 (15.3)	40	17 (42.5)	20 (50.0)	3 (7.5)
Cognitive function (%)	63	25 (39.7)	18 (28.6)	20 (31.7)	41	20 (48.8)	12 (29.3)	9 (22.0)
Social function (%)	58	22 (37.9)	16 (27.6)	20 (34.5)	39	7 (17.9)	18 (46.2)	14 (35.9)
Fatigue (%)	62	35 (56.5)	14 (22.6)	13 (21.0)	41	16 (39.0)	5 (12.2)	20 (48.8)
Nausea/vomiting (%)	64	39 (60.9)	11 (17.2)	14 (21.9)	42	24 (57.1)	5 (11.9)	13 (31.0)
Pain (%)	60	30 (50.0)	14 (23.3)	16 (26.7)	39	16 (41.0)	10 (25.6)	13 (33.3)
Dyspnoea (%)	62	33 (53.2)	7 (11.3)	22 (35.5)	41	19 (46.3)	4 (9.8)	18 (43.9)
Insomnia (%)	64	31 (48.4)	9 (14.1)	24 (37.5)	41	21 (51.2)	3 (7.3)	17 (41.5)
Appetite loss (%)	64	36 (56.3)	11 (17.2)	17 (26.6)	42	27 (64.3)	2 (4.8)	13 (31.0)
Constipation (%)	64	42 (65.6)	11 (17.2)	11 (17.2)	42	30 (71.4)	3 (7.1)	9 (21.4)
Diarrhoea (%)	64	48 (75.0)	6 (9.4)	10 (15.6)	41	30 (73.2)	2 (4.9)	9 (22.0)
Financial problems (%)	59	45 (76.3)	9 (15.3)	5 (8.5)	39	26 (66.7)	8 (20.5)	5 (12.8)

TOI = treatment outcome index

Table S5. Mean and median changes in QOL-E and EORTC QLQ-C30 domains from diagnosis to randomization

Domain	After first induction			After consolidation			
QOL-E	N	Mean change (95% CI)	Median change (95% CI)	N	Mean change (95% CI)	Median change (95% CI)	MCID
Physical	59	-3.81 (from -10.56 to 2.93)	0.00 (from 0.00 to 0.00)	39	10.26 (from 2.49 to 18.03)	12.50 (from 0.00 to 12.50)	12.30
Functional	52	0.00 (from -12.20 to 12.20)	0.00 (from 0.00 to 0.00)	37	16.52 (from 2.15 to 30.88)	11.11 (from 0.00 to 44.16)	16.52
Social	51	-2.21 (from -11.91 to 7.50)	0.00 (from -12.50 to 0.00)	35	16.07 (from 4.17 to 27.97)	12.50 (from 0.00 to 25.00)	15.43
Sexual	28	3.41 (from -7.97 to 14.79)	0.00 (from 0.00 to 8.33)	14	21.43 (from 5.45 to 37.41)	12.50 (from 0.00 to 41.67)	18.02
Fatigue	57	-1.09 (from -6.58 to 4.41)	-4.76 (from -4.76 to 0.00)	40	12.14 (from 5.87 to 18.42)	9.52 (from 4.76 to 14.28)	10.31
Disease specific	42	5.04 (from -2.59 to 12.68)	1.19 (from -2.38 to 8.33)	28	18.79 (from 10.15 to 27.43)	15.47 (from 5,95 to 26,19)	12.57
General	14	-0.27 (from -7.54 to 6.99)	-0.34 (from 9.29 to 6.07)	11	13.36 (from -3.20 to 29.92)	11.90 (from -1,46 to 22.30)	10.29
All	13	-0.09 (from -8.04 to 7.85)	1.30 (from -10.91 to 5.39)	9	13.11 (from -6.94 to 33.16)	8.00 (from -5,19 to 20,83)	10.23
TOI	34	1.76 (from -6.96 to 10.48)	4.30 (from -4.10 to 7.49)	22	15.08 (from 5.08 to 25.07)	17.52 (from 6,54 to 20.50)	11.66
EORTC QLQ-C30							
Global Health Status	63	5.55 (from -0.43 to 11.54)	8.33 (from 0.00 to 16.66)	42	18.45 (from 11.20 to 25.70)	16.66 (from 8,33 to 25.00)	13.07
Physical function	58	-9.77 (from -16.23 to -3.31)	-6.66 (from -13,33 to 0.00)	41	7.32 (from -0.28 to 14.91)	6.67 (from 0.00 to 13.33)	11.12
Role function	63	-8.02 (from -17.97 to 1.93)	0.00 (from 0.00 to -16.66)	39	8.55 (from -2.94 to 20.03)	0.00 (from 0.00 to 16.66)	15.60
Emotional function	59	1.55 (from -3.41 to 6.52)	0.00 (from 0.00 to 8.33)	40	11.67 (from 6.07 to 17.26)	12.50 (from 8,33 to 16.66)	10.08
Cognitive function	63	-0.26 (from -4.69 to 4.16)	0.00 (from 0.00 to 0.00)	41	5.28 (from -1.72 to 12.27)	0.00 (from 0.00 to 0.00)	10.75
Social function	58	-1.15 (from -8.84 to 6.54)	0.00 (from 0.00 to 0.00)	39	7.26 (from -3.09 to 17.62)	0.00 (from -16,66 to 33.33)	13.89
Fatigue	62	2.87 (from -4.32 to 10.05)	0.00 (from 0.00 to 11.11)	41	-14.63 (from -23.13 to -6.14)	-11.11 (from -22.22 to 0.00)	13.56
Nausea/vomiting	64	-0.52 (from -5.44 to 4.40)	0.00 (from 0.00 to 0.00)	42	-4.76 (from -11.86 to 2.33)	0.00 (from 0.00 to 0.00)	7.53
Pain	60	-2.78 (from -9.27 to 3.72)	0.00 (from 0.00 to 0.00)	39	-6.41 (from -14.69 to 1.87)	0.00 (from 0.00 to 0.00)	13.52
Dyspnoea	62	-10.21 (from -17.97 to -2.46)	0.00 (from 0.00 to 0.00)	41	-17.89 (from -27.89 to -7.88)	0.00 (from -33.33 to 0.00)	16.20
Insomnia	64	-7.81 (from -16.30 to 0.68)	0.00 (from -16.66 to 0.00)	41	-15.44 (from -25.17 to -5.72)	0.00 (from -33.33 to 0.00)	14.71
Appetite loss	64	-4.69 (from -12,66 to 3.28)	0.00 (from 0.00 to 0.00)	42	-13.49 (from -24.27 to -2.72)	0.00 (from 0.00 to 0.00)	15.03

Constipation	64	-3.12 (from -11.48 to 5.23)	0.00 (from 0.00 to 0.00)	42	-2.38 (from -12.99 to 8.23)	0.00 (from 0.00 to 0.00)	15.76
Diarrhoea	64	-3.12 (from -7.98 to 1.73)	0.00 (from 0.00 to 0.00)	41	-8.13 (from -15.10 to -1.16)	0.00 (from 0.00 to 0.00)	10.76
Financial problems	59	3.39 (from -1.63 to 8.41)	0.00 (from 0.00 to 0.00)	39	1.70 (from -5.28 to 8.70)	0.00 (from 0.00 to 0.00)	9.03

MCID = minimal clinically importance difference, TOI = treatment outcome index

Table S6. Linear mixed model analyses of PRO measures quality of life after randomization

Domain	Crude effect of AZA vs BSC (Regression coefficient and 95% CI)	P-value	Age-adjusted effect of AZA vs BSC (Regression coefficient and 95% CI)	P-value
QOL-E				
Physical	5.61 (from -7.20 to 18.42)	0.38	2.75 (from -9.62 to 15.11)	0.66
Function	8.67 (from -9.82 to 27.17)	0.35	6.21 (from – 11.40 to 23.80)	0.48
Social	5.57 (from -12.57 to 23.72)	0.54	4.61 (from -13.63 to 22.85)	0.61
Sexual	-8.79 (from -27.75 to 10.17	0.35	-8.64 (from -27.87 to 10.58)	0.37
Fatigue	1.94 (from -4.96 to 8.83)	0.58	0.99 (from -6.46 to 8.45)	0.79
Disease Specific	1.48 (from -8.8 to 11.78)	0.77	1.24 (from – 8.98 to 11.46)	0.81
General	5.18 (from -8.05 to 18.42)	0.43	3.66 (from – 9.33 to 16.65)	0.57
Qol Genv	9.40 (from -3.48 to 22.28)	0.15	7.49 (from -5.10 to 20.07)	0.24
All	3.49 (from -8.70 to 15.67)	0.56	2.41 (from – 9.89 to 14.72)	0.69
ALLV	7.32 (from -4.46 to 19.10)	0.22	6.15 (from -5.63 to 17.93)	0.30
TOI	9.98 (from -2.65 to 22.61)	0.12	7.92 (from -4.14 to 19.99)	0.19
EORTC QLQ-C30				
Global Health Status	-2.60 (from -13.18 to 7.98)	0.62	-2.57 (from -13.46 to 8.32)	0.64
Physical function	0.88 (from -8.84 to 10.63)	0.86	-0.91 (from -10.32 to 8.41)	0.84
Role function	1.58 (from -6.45 to 9.61)	0.70	1.60 (from -6.38 to 9.58	0.69
Emotional function	-0.04 (from -6.67 to 6.60)	0.99	0.18 (from -6.59 to 6.93)	0.96
Cognitive function	1.72 (from -3.62 to 7.06)	0.53	1.91 (from -4.33 to 8.16)	0.54
Social function	5.17 (from -5.34 to 15.66)	0.32	4.20 (from -6.47 to 14.88)	0.43
Fatigue	3.98 (from -4.60 to 12.55)	0.36	4.27 (from -4.15 to 12.69)	0.31
Nausea/vomiting	-0.33 (from -3.98 to 3.32)	0.86	1.00 (from -4.40 to 2.40)	0.56
Pain	-2.50 (from -12.79 to 7.78)	0.62	-2.24 (from -12.83 to 8.35)	0.67
Dyspnoea	-5.53 (from -14.04 to 2.98)	0.20	-5.06 (from -13.61 to 3.49)	0.24
Insomnia	-2.93 (from -0.73 to 4.85)	0.45	-2.92 (from -10.89 to 5.06)	0.46
Appetite loss	0.81 (from -4.46 to 6.07)	0.76	0.17 (from -4.79 to 5.12)	0.95
Constipation	5.15 (from -6.02 to 16.33)	0.36	5.57 (from -5.76 to 16.90)	0.33
Diarrhoea	-3.24 (from -9.70 to 3.23)	0.32	-3.07 (from -9.68 to 3.52)	0.36
Financial problems	-3.02 (from -9.74 to 3.70)	0.37	0.28 (from -7.11 to 7.68)	0.94

TOI = treatment outcome index

Table S7. Generalised estimating equation of PRO measures after randomization

	Crude effect of AZA vs BSC (Odds ratio and 95% CI)	P-value	Age-adjusted effect of AZA vs BSC (Odds ratio and 95% CI)	P-value
QOL-E				
MCID Physical	0.64 (from 0.18 to 2.28)	0.50	0.66 (from 0.19 to 2.32)	0.52
MCID Function	1.17 (from 0.29 to 4.81)	0.83	1.17 (from 0.29 to 4.73)	0.82
MCID Social	0.69 (from 0.11 to 4.29)	0.69	0.80 (from 0.13 to 4.73)	0.80
MCID Sexual	0.46 (from 0.08 to 2.65)	0.38	0.47 (from 0.08 to 2.58)	0.38
MCID Fatigue	0.54 (from 0.14 to 2.13)	0.38	0.63 (from 0.16 to 2.48)	0.51
MCID Disease Specific	0.56 (from 0.14 to 2.29)	0.42	0.56 (from 0.14 to 2.28)	0.42
MCID General	0.67 (from 0.09 to 5.12)	0.70	0.22 (from 0.03 to 1.52)	0.13
MCID All	0.64 (from 0.08 to 5.36)	0.68	0.47 (from 0.05 to 4.16)	0.49
*MCID TOI				
EORTC QLQ-C30				
MCID Global Health Status	0.94 (from 0.22 to 4.12)	0.94	0.96 (from 0.21 to 4.32)	0.96
MCID Physical function	0.63 (from 0,18 to 2.14)	0.46	0.64 (from 0.18 to 2.31)	0.50
MCID Role function				
MCID Emotional function	2.77 (from 0.14 to 55.8)	0.51	1.73 (from 0.08 to 39.4)	0.73
MCID Cognitive function	0.42 (from 0.11 to 1.69)	0.22	0.43 (from 0.11 to 1.76)	0.24
MCID Social function	1.44 (from 0.34 to 6.02)	0.62	1.69 (from 0.44 to 6.47)	0.44
MCID Fatigue	0.71 (from 0.21 to 2.43)	0.59	0.73 (from 0.21 to 2.61)	0.63
MCID Nausea/vomiting	0.79 (from 0.15 to 4.08)	0.78	0.44 (from 0.07 to 2.89)	0.40
MCID Pain	1.15 (from 0.35 to 3.77)	0.82	1.03 (from 0.32 to 3.34)	0.96
MCID Dyspnoea	1.29 (from 0.44 to 4.17)	0.67	1.22 (from 0.37 to 3.99)	0.74
MCID Insomnia	0.87 (from 0.26 to 2.87)	0.82	0.86 (from 0.25 to 2.88)	0.80
MCID Appetite loss	0.54 (from 0.11 to 2.60)	0.45	0.43 (from 0.09 to 2.15)	0.31
MCID Constipation	8.55 (from 0.87 to 84.09)	0.08	7.85 (from 0.77 to 79.52)	0.08
MCID Diarrhoea	0.41 (from 0.09 to 1.78)	0.23	0.29 (from 0.06 to 1.44)	0.13
MCID Financial problems	2.00 (from 0.47 to 8.65)	0.35	1.57 (from 0.36 to 6.88)	0.55

*Convergence was not achieved for this domain. MCID = minimal clinically importance difference, TOI = treatment outcome index

Table S8. The MCIDs and changes in PRO scores in the first 6 months following randomization according to allocation arm

Domain		Second month post remission			Fourth month post remission			Sixth month post remission			
QOL-E	Arm	N	Mean change (95% CI)	Median change (95% CI)	N	Mean change (95% CI)	Median change (95% CI)	N	Mean change (95% CI)	Median change (95% CI)	MCID
Physical	BSC	9	4.17 (from -6.57 to 14.91)	0.00 (from -12.50 to 25.00)	10	8.75 (from -4.61 to 22.11)	0.00 (from -6.25 to 25.00)	6	6.25 (from -11.83 to 24.33)	0.00 (from -6.25 to 25.00)	12.30
	AZA	15	-0.83 (from -8.88 to 7.22)	0.00 (from -12.18 to 12.50)	13	6.73 (from -4.22 to 17.68)	0.00 (from 0.00 to 12.50)	10	8.75 (from -3.93 to 21.43)	12.50 (from -12.50 to 25.00)	
Functional	BSC	9	4.93 (from -9.92 to 19.80)	0.00 (from 0.00 to 0.00)	12	2.78 (from -4.04 to 9.59)	0.00 (from 0.00 to 5.55)	7	9.52 (from -18.57 to 37.62)	0.00 (from 0.00 to 0.00)	16.52
	AZA	15	5.18 (from -10.74 to 21.11)	0.00 (from 0.00 to 11.11)	12	5.55 (from -15.84 to 26.95)	0.00 (from 0.00 to 2.22)	8	4.17 (from -28.74 to 37.08)	0.00 (from -11.11 to 44.44)	
Social	BSC	11	-4.54 (from -13.17 to 4.08)	0.00 (from -12.50 to 0.00)	12	-26.04 (from -51.26 to 0.82)	-6.25 (from -43.75 to 0.00)	7	-12.50 (from -50.84 to 25.84)	0.00 (from -25.00 to 12.50)	15.43
	AZA	15	-12.50 (from -23.60 to -1.40)	0.00 (from -25.00 to 0.00)	10	-15.00 (from -37.62 to 7.62)	0.00 (from -50.00 to 0.00)	5	-12.50 (from -39.38 to 14.38)	0.00 (from -50.00 to 0.00)	
Sexual	BSC	4	10.42 (from -9.47 to 30.31)	8.33 (from 0.00 to 25.00)	5	16.67 (from -11.67 to 45.00)	0.00 (from 0.00 to 41.66)	4	22.92 (from -33.73 to 79.56)	8.33 (from 0.00 to 75.00)	18.02
	AZA	7	-11.90 (from -31.22 to 7.42)	0.0 (from -33.33 to 0.00)	7	-16.67 (from -48.44 to 15.11)	-16.66 (from -50.00 to 0.00)	4	-33.33 (from -75.27 to 8.60)	-37.50 (from -58.33 to 0.00)	
Fatigue	BSC	11	0.0 (from -4.52 to 4.52)	0.00 (from 0.00 to 0.00)	12	0.39 (from -5.14 to 5.94)	0.00 (from -4.76 to 4.76)	7	-1.36 (from -12.06 to 9.34)	0.00 (from -14,28 to 4,76)	10.31
	AZA	16	-0.59 (from -5.74 to 4.55)	0.00 (from -2.38 to 4.76)	12	-0.79 (from -6.54 to 4.95)	0.00 (from -7.14 to 4.76)	8	-2.38 (from -10.34 to 5.58)	0.00 (from -4,76 to 4,76)	
Disease Specific	BSC	10	10.71 (from -3.47 to 24.90)	7.14 (from -3,57 to 29,76)	11	5.63 (from -9.53 to 20.79)	4.76 (from -14.28 to 21.42)	7	2.38 (from -19.56 to 24.33)	0.00 (from -19,05 to 28.57)	12.57
	AZA	12	-1.78 (from -8.55 to 4.98)	-3.57 (from -9,52 to 7,14)	9	-2.64 (from -13.68 to 8.39)	0.00 (from -14.28 to 9.52)	4	-3.57 (from -10.13 to 2.99)	-3.57 (from -7.14 to 0.00)	

General	BSC	4	2.34 (from -15.54 to 20.22)	5.00 (from -12.57 to 11,94)	5	-1.10 (from -13.52 to 11.32)	2.50 (from -12,57 to 14.68)	4	7.92 (from -27.29 to 43.13)	2.44 (from -12.57 to 39.36)	10.29
	AZA	5	-8.6 (from -19.81 to 2.54)	-4.32 (from -20,55 to 0.00)	4	1.90 (from -17.98 to 21.79)	3.39 (from -13,45 to 14,28)	1	-10.12	-10.12	
All	BSC	3	0.35 (from -35.88 to 36.59)	0.00 (from -14.05 to 15,11)	4	-0.08 (from -23.44 to 23.27)	-3.42 (from -14,05 to 20.56)	4	7.59 (from -28.17 to 43.34)	2.62 (from -14,05 to 39.15)	10.23
	AZA	4	-6.29 (from -20.70 to 8.10)	-2.84 (from -19,51 to 0.00)	4	2.88 (from -14.36 to 20.11)	3.42 (from -9.62 to 14.28)	1	-9.63	-9.63	
TOI	BSC	7	4.92 (from -8.04 to 17.88)	0.00 (from -2.38 to 14,68)	9	5.33 (from -5.92 to 16.58)	2.38 (from -8.92 to 18.38)	6	5.13 (from -14.06 to 24.31)	3.07 (from -10.28 to 22.58)	11.66
	AZA	9	2.64 (from -6.42 to 11.70)	0.00 (from -2.84 to 8.46)	7	6.09 (from -5.04 to 17.22)	1.78 (from 0.00 to 4.76)	3	12.48 (from -20.46 to 45.42)	8.33 (from 1.78 to 27.31)	
EORTC QLQ-C30											
Global Health Status	BSC	11	4.54 (from -5.58 to 14.67)	0.00 (from 0.00 to 16.66)	11	-8.33 (from -29.42 to 12.76)	0.00 (from -25.00 to 16.67)	7	2.38 (from -21.87 to 26.64)	0.00 (from -16.67 to 16.67)	13.07
	AZA	18	0.92 (from -4.74 to 6.59)	0.00 (from -4.17 to 8.33)	15	-0.56 (from -6.96 to 5.84)	0.00 (from -8.33 to 8.33)	10	1.66 (from -9.14 to 12.50)	0.00 (from -8.33 to 8.33)	
Physical function	BSC	11	3.03 (from -2.40 to 8.46)	6.66 (from 0.00 to 6.66)	12	2.22 (from -4.12 to 8.56)	0.00 (from -6.67 to 10.00)	7	5.71 (from -12.24 to 23.66)	0.00 (from -6.67 to 6.67)	11.12
	AZA	17	-3.14 (from -9.45 to 3.17)	0.00 (from -6.66 to 0.00)	14	-1.43 (from -7.68 to 4.83)	0.00 (from -6.67 to 6.67)	9	0.00 (from -8.10 to 8.10)	0.00 (from -13.33 to 6.67)	
Role function	BSC	10	3.33 (from -8.98 to 15.65)	0.00 (from 0.00 to 16.66)	12	2.78 (from -12.08 to 17.64)	0.00 (from -16.67 to 16.678)	7	7.14 (from -31.50 to 45.79)	0.00 (from -33.33 to 33.33)	15.60
	AZA	18	13.89 (from -3.94 to 23.84)	0.00 (from 0.00 to 33.33)	14	4.76 (from -5.52 to 15.05)	0.00 (from 0.00 to 16.67)	10	10.00 (from -13.98 to 33.98)	0.00 (from -8.33 to 33.33)	
Emotional function	BSC	11	3.79 (from -12.23 to 4.65)	0.00 (from -16.66 to 8.33)	12	-6.94 (from -20.65 to 6.76)	0.00 (from -12.50 to 8.33)	7	-4.76 (from -16.41 to 6.89)	0.00 (from -16.67 to 8.33)	10.08
	AZA	18	-0.46 (from -5.87 to 4.94)	0.00 (from 0.00 to 4.17)	15	-1.67 (from -9.31 to 5.98)	0.00 (from -8.33 to 8.33)	10	0.00 (from -13.18 to 13.18)	0.00 (from -8.33 to 8.33)	
Cognitive function	BSC	11	1.51 (from -12.16 to 15.19)	0.00 (from 0.00 to 16.66)	12	0.00 (from -11.06 to 11.06)	0.00 (from 0.00 to 16.67)	7	0.00 (from -21.80 to 21.80)	0.00 (from 0.00 to 16.67)	10.75
	AZA	18	-2.78 (from -8.64 to 3.08)	0.00 (from 0.00 to 0.00)	15	-2.22 (from -8.13 to 3.68)	0.00 (from -16.24 to 0.00)	10	-6.67 (from -20.66 to 7.33)	0.00 (from -16.67 to 8.33)	

Social function	BSC	11	0.00 (from -5.01 to 5.01)	0.00 (from 0.00 to 0.00)	12	-5.55 (from -22.65 to 11.54)	0.00 (from 0.00 to 0.00)	7	11.90 (from -5.25 to 29.06)	0.00 (from 0.00 to 16.67)	13.89
	AZA	18	3.70 (from -3.00 to 10.40)	0.00 (from 0.00 to 0.00)	15	4.44 (from -5.09 to 13.98)	0.00 (from 0.00 to 16.67)	10	-1.67 (from -17.01 to 13.67)	0.00 (from -25.00 to -16.67)	
Fatigue	BSC	10	2.22 (from -8.24 to 12.69)	0.00 (from -11.11 to 22.22)	11	2.02 (from 8.44 to 12.48)	0.00 (from -11.11 to 11.11)	7	-4.76 (from -33.13 to 23.60)	0.00 (from -11.11 to 22.22)	13.56
	AZA	19	1.17 (from -9.22 to 11.56)	0.00 (from 0.00 to 11.11)	15	4.44 (from -5.37 to 14.26)	0.00 (from -11.11 to 11.11)	11	-4.04 (from -20.49 to 12.41)	0.00 (from -11.11 to 11.11)	
Nausea/vomiting	BSC	11	-3.03 (from -7.56 to 1.50)	0.00 (from 0.00 to 0.00)	12	0.00 (from -4.51 to 4.51)	0.00 (from 0.00 to 0.00)	7	0.00 (from -8.90 to 8.90)	0.00 (from 0.00 to 0.00)	7.53
	AZA	18	-2.78 (from -13.12 to 7.57)	0.00 (from 0.00 to 0.00)	15	1.11 (from -1.27 to 3.49)	0.00 (from 0.00 to 0.00)	11	1.51 (from -21.68 to 24.71)	0.00 (from 0.00 to 16.67)	
Pain	BSC	11	-6.06 (from -19.56 to 7.44)	0.00 (from -16.66 to 0.00)	12	2.78 (from -8.13 to 13.68)	0.00 (from 0.00 to 16.67)	7	4.76 (from -9.90 to 19.42)	0.00 (from 0.00 to 16.67)	13.52
	AZA	18	0.93 (from -4.37 to 6.22)	0.00 (from 0.00 to 0.00)	15	3.33 (from -5.35 to 12.02)	0.00 (from -16.67 to 16.67)	9	16. 67 (from -8.96 to 42.29)	16.67 (from 0.00 to 16.67)	
Dyspnoea	BSC	11	3.03 (from -3.72 to 9.78)	0.00 (from 0.00 to 0.00)	12	0.00 (from -15.64 to 15.64)	0.00 (from -16.67 to 16.67)	7	4.76 (from -16.51 to 26.03)	0.00 (from 0.00 to 33.33)	16.20
	AZA	18	11.11 (from 1.26 to 20.96)	0.00 (from 0.00 to 16.66)	15	13.33 (from -1.66 to 25.01)	0.00 (from 0.00 to 33.33)	10	10.00 (from -1.52 to 21.52)	0.00 (from 0.00 to 33.33)	
Insomnia	BSC	11	0.0 (from -10.01 to 10.01)	0.00 (from 0.00 to 0.00)	12	8.33 (from -15.77 to 32.44)	0.00 (from 0.00 to 16.67)	7	0.00 (from -30.83 to 30.83)	0.00 (from 0.00 to 33.33)	14.71
	AZA	18	0.0 (from -5.68 to 5.68)	0.00 (from 0.00 to 0.00)	15	6.67 (from -5.81 to 19.15)	0.00 (from 0.00 to 33.33)	10	10.00 (from -1.52 to 21.52)	0.00 (from 0.00 to 33.33)	
Appetite loss	BSC	11	-6.06 (from -19.56 to 7.44)	0.00 (from -33.33 to 0.00)	12	-5.55 (from -17.78 to 6.67)	0.00 (from 16.67 to 0.00)	7	-9.52 (from -32.83 to 13.78)	0.00 (from -33.33 to 0.00)	15.03
	AZA	19	-5.26 (from -17.55 to 7.02)	0.00 (from 0.00 to 0.00)	15	0.0 (from -6.98 to 6.98)	0.00 (from 0.00 to 0.00)	11	-9.09 (from -35.76 to 17.58)	0.00 (from -33.33 to 0.00)	
Constipation	BSC	11	-12.12 (from -32.82 to 8.58)	0.00 (from 0.00 to 0.00)	12	8.33 (from -21.50 to 4.83)	0.00 (from 0.00 to 0.00)	7	-28.57 (from -61.53 to 4.38)	-33.33 (from -33.33 to 0.00)	15.76
	AZA	19	-3.51 (from -21.18 to 14.17)	0.00 (from -33.33 to 0.00)	15	-6.67 (from -19.15 to 5.81)	0.00 (from -33.33 to 0.00)	11	-12.12 (from -	0.00 (from -33.33 to 0.00)	

									35.12 to 10.87)		
Diarrhoea	BSC	11	-3.03 (from -15.11 to 9.05)	0.00 (from 0.00 to 0.00)	12	8.33 (from -7.63 to 24.30)	0.00 (from 0.00 to 16.67)	7	4.76 (from -16.52 to 26.03)	0.00 (from 0.00 to 33.33)	10.76
	AZA	18	1.85 (from -2.05 to 5.76)	0.00 (from 0.00 to 0.00)	15	4.44 (from -2.05 to 10.94)	0.00 (from 0.00 to 0.00)	10	3.33 (from -4.21 to 10.87)	0.00 (from 0.00 to 0.00)	
Financial problems	BSC	11	-3.03 (from -9.78 to 3.72)	0.00 (from 0.00 to 0.00)	12	8.33 (from -4.83 to 21.50)	0.00 (from 0.00 to 0.00)	7	-4.76 (from -16.41 to 6.89)	0.00 (from 0.00 to 0.00)	9.03
	AZA	18	-3.70 (from -13.37 to 5.96)	0.00 (from 0.00 to 0.00)	15	2.22 (from -8.74 to 13.18)	0.00 (from 0.00 to 0.00)	10	10.00 (from -15.26 to 35.26)	0.00 (from 0.00 to 16.67)	

MCID = minimal clinically importance difference, TOI = treatment outcome index

Table S9. The proportions of patients who remained stable, improved, or worsened according to the MCID after randomization

Domain		Second month post remission				Fourth month post remission				Sixth month post remission			
QOL-E		N	Stable	Improved	Worsened	N	Stable	Improved	Worsened	N	Stable	Improved	Worsened
Physical (%)	BSC	9	4 (44.4)	3 (33.3)	2 (22.2)	10	4 (40.0)	4 (40.0)	2 (20.0)	6	3 (50.0)	2 (33.3)	1 (16.7)
	AZA	15	7 (46.7)	4 (26.7)	4 (26.7)	13	5 (38.5)	5 (38.5)	3 (23.1)	10	1 (10.0)	6 (60.0)	3 (30.0)
Functional (%)	BSC	9	8 (88.9)	1 (11.1)	-	12	10 (83.3)	2 (16.7)	-	7	6 (85.7)	1 (14.3)	-
	AZA	15	10 (66.7)	3 (20.0)	2 (13.3)	12	8 (66.7)	3 (25.0)	1 (8.3)	8	5 (62.5)	2 (25.0)	1 (25.5)
Social (%)	BSC	11	9 (81.8)	-	2 (16.2)	12	7 (58.3)	-	5 (41.7)	7	4 (57.1)	1 (14.3)	2 (28.6)
	AZA	15	8 (53.3)	1 (6.7)	6 (40.0)	10	6 (60.0)	1 (10.0)	3 (30.0)	5	4 (80.0)	-	1 (20.0)
Sexual (%)	BSC	4	3 (75.0)	1 (3.7)	-	5	3 (60.0)	2 (40.0)	-	4	3 (75.0)	1 (25.0)	-
	AZA	7	5 (71.4)	-	2 (28.6)	7	3 (42.9)	1 (14.3)	3 (42.9)	4	1 (25.0)	-	3 (75.0)
Fatigue (%)	BSC	11	9 (81.8)	1 (9.1)	1 (9.1)	12	9 (75.0)	2 (16.7)	1 (8.3)	7	4 (57.1)	1 (14.3)	2 (28.6)
	AZA	16	14 (87.5)	1 (6.3)	1 (6.3)	12	9 (75.0)	1 (8.3)	2 (16.7)	8	7 (87.5)	-	1 (12.5)
Disease Specific (%)	BSC	10	4 (40.0)	5 (50.0)	1 (10.0)	11	3 (27.3)	4 (36.4)	4 (36.4)	7	2 (28.6)	2 (28.6)	3 (42.9)
	AZA	12	8 (66.7)	2 (7.4)	2 (7.4)	9	6 (66.7)	1 (11.1)	2 (22.2)	4	4 (100.0)	-	-
General (%)	BSC	4	2 (50.0)	1 (25.0)	1 (25.0)	5	3 (60.0)	1 (20.0)	1 (20.0)	4	2 (50.0)	1 (25.0)	1 (25.0)
	AZA	5	3 (60.0)	-	2 (40.0)	4	2 (50.0)	1 (25.0)	1 (25.0)	1	1 (100.0)	-	-
All (%)	BSC	3	1 (33.3)	1 (33.3)	1 (33.3)	4	2 (50.0)	1 (25.0)	1 (25.0)	4	2 (50.0)	1 (25.0)	1 (25.0)
	AZA	4	3 (75.0)	-	1 (25.0)	4	3 (75.0)	1 (25.0)	-	1	1 (100.0)		
TOI (%)	BSC	7	4 (57.1)	2 (28.6)	1 (14.3)	9	5 (55.6)	3 (33.3)	1 (11.1)	6	4 (66.7)	1 (16.7)	1 (16.7)
	AZA	9	7 (77.8)	1 (11.1)	1 (11.1)	7	6 (85.7)	1 (14.3)	-	3	2 (66.7)	1 (33.3)	-
EORTC QLQ-C30													
Global Health Status (%)	BSC	11	5 (45.5)	4 (36.4)	2 (18.2)	11	4 (36.4)	3 (27.3)	4 (36.4)	7	3 (42.9)	2 (28.6)	2 (28.6)
	AZA	18	11 (61.1)	4 (22.2)	3 (16.7)	15	9 (60.0)	3 (20.0)	3 (20.0)	10	6 (60.0)	2 (20.0)	2 (20.0)
Physical function (%)	BSC	11	8 (72.7)	2 (18.2)	1 (9.1)	12	8 (66.7)	3 (25.0)	1 (8.3)	7	5 (71.4)	1 (14.3)	1 (14.3)
	AZA	17	11 (64.7)	2 (11.8)	4 (23.5)	14	10 (71.4)	1 (7.1)	3 (21.4)	9	6 (66.7)	1 (11.1)	2 (22.2)
Role function (%)	BSC	10	6 (60.0)	3 (30.0)	1 (10.0)	12	4 (33.3)	4 (33.3)	4 (33.3)	7	1 (14.3)	3 (42.9)	3 (42.9)

	AZA	18	11 (61.1)	7 (38.9)	-	14	7 (50.0)	5 (35.7)	2 (14.3)	10	4 (40.0)	4 (40.0)	2 (20.0)
Emotional function (%)	BSC	11	8 (72.7)	-	3 (27.3)	12	9 (75.0)	-	3 (25.0)	7	5 (71.4)	-	2 (28.6)
	AZA	18	15 (83.3)	1 (5.6)	2 (11.1)	15	12 (80.0)	1 (6.7)	2 (13.3)	10	6 (60.0)	2 (20.0)	2 (20.0)
Cognitive function (%)	BSC	11	5 (45.5)	4 (36.4)	2 (18.2)	12	6 (50.0)	4 (33.3)	2 (16.7)	7	3 (42.9)	3 (42.9)	1 (14.3)
	AZA	18	12 (66.7)	2 (11.1)	4 (22.2)	15	9 (60.0)	2 (13.3)	4 (26.7)	10	4 (40.0)	2 (20.0)	4 (40.0)
Social function (%)	BSC	11	9 (81.8)	1 (9.1)	1 (9.1)	12	8 (66.7)	2 (16.7)	2(16.7)	7	4 (57.1)	3 (42.9)	-
	AZA	18	12 (66.7)	4 (14.8)	2 (11.1)	15	8 (53.3)	5 (33.3)	2 (13.3)	10	4 (40.0)	3 (30.0)	3 (30.0)
Fatigue (%)	BSC	10	7 (70.0)	3 (30.0)	-	11	8 (72.7)	2 (18.2)	1 (9.1)	7	4 (57.1)	2 (28.6)	1 (14.3)
	AZA	19	13 (68.4)	4 (21.1)	2 (10.5)	15	11 (73.3)	3 (20.0)	1 (6.7)	11	7 (63.6)	2 (18.2)	2 (18.2)
Nausea/vomiting (%)	BSC	11	9 (81.8)	-	2 (18.2)	12	10 (83.3)	1 (8.3)	1 (8.3)	7	5 (71.4)	1 (14.3)	1 (14.3)
	AZA	18	15 (83.3)	2 (11.1)	1 (5.6)	15	14 (93.3)	1 (6.7)	-	11	7 (63.6)	3 (27.3)	1 (9.1)
Pain (%)	BSC	11	6 (54.5)	1 (9.1)	4 (36.4)	12	6 (50.0)	4 (33.3)	2 (16.7)	7	4 (57.1)	2 (28.6)	1 (14.3)
	AZA	18	11 (61.1)	4 (22.2)	3 (16.7)	15	5 (33.3)	6 (40.0)	4 (26.7)	9	3 (33.3)	5 (55.6)	1 (11.1)
Dyspnoea (%)	BSC	11	10 (90.9)	1 (9.1)	-	12	6 (50.0)	3 (25.0)	3 (25.0)	7	4 (57.1)	2 (28.6)	1 (14.3)
	AZA	18	13 (72.2)	5 (27.8)	-	15	10 (66.7)	5 (33.3)	-	10	7 (70.0)	3 (30.0)	-
Insomnia (%)	BSC	11	9 (81.8)	1 (9.1)	1 (9.1)	12	8 (66.7)	3 (25.0)	1 (8.3)	7	4 (57.1)	2 (28.6)	1 (14.3)
	AZA	18	16 (88.9)	1 (5.6)	1 (5.6)	15	8 (53.3)	5 (33.3)	2 (13.3)	10	7 (70.0)	3 (30.0)	-
Appetite loss (%)	BSC	11	7 (63.6)	1 (9.1)	3 (27.3)	12	8 (66.7)	1 (8.3)	3 (25.0)	7	3 (42.9)	1 (14.3)	3 (42.9)
	AZA	19	16 (84.2)	1 (5.3)	2 (10.5)	15	13 (86.7)	1 (6.7)	1 (6.7)	11	7 (63.6)	1 (9.1)	3 (27.3)
Constipation (%)	BSC	11	9 (81.8)	-	2 (18.2)	12	10 (83.3)	-	2 (16.7)	7	3 (42.9)	-	4 (57.1)
	AZA	19	8 (42.1)	5 (26.3)	6 (31.6)	15	8 (53.3)	2 (13.3)	5 (33.3)	11	7 (63.6)	1 (9.1)	3 (27.3)
Diarrhoea (%)	BSC	11	8 (72.7)	1 (9.1)	2 (18.2)	12	8 (66.7)	3 (25.0)	1 (8.3)	7	4 (57.1)	2 (28.6)	1 (14.3)
	AZA	18	17 (94.4)	1 (5.6)	-	15	13 (86.7)	2 (13.3)	-	10	9 (90.0)	1 (10.0)	-
Financial problems (%)	BSC	11	10 (90.9)	-	1 (9.1)	12	10 (83.3)	2 (16.7)	-	7	6 (85.7)	-	1 (14.3)
	AZA	18	12 (66.7)	2 (11.1)	4 (22.2)	15	10 (66.7)	3 (20.0)	2 (13.3)	10	7 (70.0)	2 (20.0)	1 (10.0)

MCID = minimal clinically importance difference, TOI = treatment outcome index
